# TSSC3 promotes autophagy via inactivating the Src-mediated PI3K/Akt/mTOR pathway to suppress tumorigenesis and metastasis in osteosarcoma, and predicts a favorable prognosis

**DOI:** 10.1186/s13046-018-0856-6

**Published:** 2018-08-09

**Authors:** Guo-sheng Zhao, Zi-ran Gao, Qiao Zhang, Xue-feng Tang, Yang-fan Lv, Zhao-si Zhang, Yuan Zhang, Qiu-lin Tan, Dong-bin Peng, Dian-ming Jiang, Qiao-Nan Guo

**Affiliations:** 1 Department of Orthopedic Surgery, The First Affiliated Hospital of Chongqing Medical University, Chongqing, 400016 People’s Republic of China; 2 Department of Pathology, Xinqiao Hospital, The Third Military Medical University, Chongqing, 400037 People’s Republic of China; 3 Department of Rehabilitation, The First Affiliated Hospital of Chongqing Medical University, Chongqing, 400016 People’s Republic of China; 4 Bone and Trauma Center, The Third Affiliated Hospital of Chongqing Medical University, Chongqing, 401120 People’s Republic of China; 5 Department of Neurosurgery, The First Affiliated Hospital of Chongqing Medical University, Chongqing, 400016 People’s Republic of China; 6 Department of Orthopaedics, Ministry of Education Key Laboratory of Child Development and Disorders, Key Laboratory of Pediatrics in Chongqing, China International Science and Technology Cooperation Base of Child Development and Critical Disorders, Children’s Hospital of Chongqing Medical University, Chongqing, 400014 People’s Republic of China

**Keywords:** TSSC3, Autophagy, Osteosarcoma, ATG5, Src, PI3K/Akt/mTOR, Tumorigenesis, Metastasis

## Abstract

**Background:**

Over the last two or three decades, the pace of development of treatments for osteosarcoma tends has been slow. Novel effective therapies for osteosarcoma are still lacking. Previously, we reported that tumor-suppressing STF cDNA 3 (*TSSC3*) functions as an imprinted tumor suppressor gene in osteosarcoma; however, the underlying mechanism by which TSSC3 suppresses the tumorigenesis and metastasis remain unclear.

**Methods:**

We investigated the dynamic expression patterns of TSSC3 and autophagy-related proteins (autophagy related 5 (ATG5) and P62) in 33 human benign bone tumors and 58 osteosarcoma tissues using immunohistochemistry. We further investigated the correlations between TSSC3 and autophagy in osteosarcoma using western blotting and transmission electronic microscopy. CCK-8, Edu, and clone formation assays; wound healing and Transwell assays; PCR; immunohistochemistry; immunofluorescence; and western blotting were used to investigated the responses in TSSC3-overexpressing osteosarcoma cell lines, and in xenografts and metastasis in vivo models, with or without autophagy deficiency caused by chloroquine or ATG5 silencing.

**Results:**

We found that ATG5 expression correlated positively with TSSC3 expression in human osteosarcoma tissues. We demonstrated that TSSC3 was an independent prognostic marker for overall survival in osteosarcoma, and positive ATG5 expression associated with positive TSSC3 expression suggested a favorable prognosis for patients. Then, we showed that TSSC3 overexpression enhanced autophagy via inactivating the Src-mediated PI3K/Akt/mTOR pathway in osteosarcoma. Further results suggested autophagy contributed to TSSC3-induced suppression of tumorigenesis and metastasis in osteosarcoma in vitro and in vivo models.

**Conclusions:**

Our findings highlighted, for the first time, the importance of autophagy as an underlying mechanism in TSSC3-induced antitumor effects in osteosarcoma. We also revealed that TSSC3-associated positive ATG5 expression might be a potential predictor of favorable prognosis in patients with osteosarcoma.

**Electronic supplementary material:**

The online version of this article (10.1186/s13046-018-0856-6) contains supplementary material, which is available to authorized users.

## Background

Osteosarcoma is the most frequent type of primary malignancy of bone and is also the second most common cause of cancer-related death in young adolescents and children [1, 2]. In recent years, given the combination of chemotherapy and surgery as a primary treatment for osteosarcoma, the five-year survival rate has increased to approximately 65–70% for no distant metastasis disease. However, despite progress of in surgical techniques and chemotherapeutic drugs, the five-year survival rate remains poor because osteosarcoma is highly malignant with highly aggressive behavior and shows resistance to chemotherapies, resulting in a five-year survival rate for early lung metastatic patients of only 20% [3, 4]. Over the last two or three decades there have been few developments in the treatment of osteosarcoma. Thus, novel effective therapies for osteosarcoma are still lacking and are urgently required.

Genomic imprinting is an epigenetic form of genetic regulation that results in monoallelic gene expression (maternal or paternal). It is widely accepted that genomic imprinting of tumor suppressor genes contributes to tumor susceptibility because mutation of only one allele is needed [5]. Meanwhile, genomic imprinting, as a form of epigenetic and reversible gene control, can be altered by epigenetic drugs, which highlights a new potential treatment of cancer. Tumor-suppressing STF cDNA 3 (*TSSC3*), also known as *PHLDA2*, *IPL* or *BWR1C*, is located on chromosome 11p15, a tumor suppressor region, and is the first identified apoptosis-related imprinted gene [6]. Our group revealed that *TSSC3* expression is downregulated in osteosarcoma cells, suggesting it as a promising therapeutic target for osteosarcoma [7]. Furthermore, we demonstrated that *TSSC3* functions as a tumor suppressor gene, inducing apoptosis, and suppressing tumorigenesis and metastasis in osteosarcoma, and is associated with favorable overall survival (OS) [4, 8–11]. Despite these findings, the underlying mechanism by which TSSC3 suppresses tumorigenesis and metastasis in osteosarcoma is incompletely understood.

Autophagy is an essential and highly conserved cellular process that targets selective proteins and abnormal organelles for lysosomal degradation [12]. The role of autophagy in cancer is controversial. On the one hand, autophagy can function as a cytoprotective response to chemotherapeutic drugs in cancer cells and promotes metastasis through facilitating the mobility and anoikis resistance of tumor cells [13–17]. On the other hand, numerous studies have demonstrated that autophagy can induce autophagic cell death, cell proliferation inhibition, and oncoproteins degradation to suppresses tumorigenesis, impede metastasis, and even enhance chemosensitivity [18–23]. Recently, it has been revealed that autophagy could be regulated by imprinted genes in some cancer cells, such as ovarian cancer cells [20] and bladder cancer cells [24]. However, the connection between autophagy and imprinted gene in osteosarcoma is less explored. More recently, *PHLDA1*, the homologous gene of *TSSC3* has been demonstrated to trigger autophagy [25]. Moreover, the PI3K/Akt/mTOR signaling pathway, which is a classical pathway that modulates cell proliferation, apoptosis resistance, and tumorigenesis, is reported to be involved in the regulation of autophagy in several human tumors cells [26, 27] and can be activated by the Src-family kinases [28]. Our previous studies found that TSSC3 could inhibit the phosphorylation of both Src and Akt in osteosarcoma cells [10, 11]; therefore, we speculated that autophagy might be involved in the anti-tumor effect of TSSC3.

In the present study, we investigated the correlation between TSSC3 and autophagy-related gene 5 (*ATG5*) protein expression (one of the key proteins for the formation of autophagosomes) [29] in human osteosarcoma tissues and their prognostic value in osteosarcoma. Furthermore, we observed enhanced autophagy flux in TSSC3 overexpressing osteosarcoma cells and demonstrated that TSSC3-induced impairment of tumorigenesis and metastasis in osteosarcoma cells was reduced when autophagy was inhibited using chloroquine (CQ) or under conditions of stable knockdown of *ATG5* by lentiviral vectors in vitro *and* in vivo. In addition, the Src-mediated PI3K/Akt/mTOR signaling pathway was found to be involved in TSSC3-induced autophagy. To the best of our knowledge, no previous study has demonstrated the correlation between TSSC3 and autophagy. The findings of this study provide novel insights into the underlying mechanism by which TSSC3 suppresses tumorigenesis and metastasis in osteosarcoma by highlighting the role of autophagy.

## Methods

### Human specimens

Specimens were obtained from 58 patients with histopathologically confirmed osteosarcoma with no preoperative anticancer treatment from Southwest Hospital and Xinqiao Hospital, Third Military Medical University (TMMU), Chongqing, China between February 2011 and November 2015. The last follow-up time was November 2017. The clinicopathological features of the patients are listed in Table 1. Two certified pathologists classified all the specimens as high-grade osteosarcoma. Lung metastasis and local recurrence were diagnosed by both imaging and pathology. The surgical margins and stage were classified according to the Enneking system. Patients with primary osteosarcoma were classified as with or without developed distant metastasis at diagnosis or after surgery. Written informed consent for the experimental studies was obtained from the patients or their guardians. All experiments were approved by the Institutional Ethics Committee of TMMU.

**Table 1:** Correlations between TSSC3, ATG5, and P62 expression and the clinicopathological features of osteosarcoma

Clinical characteristics	Group	n	TSSC3		*P*-value^#^	ATG5		*P*-value^#^	P62		*P*-value^#^
			Positive	negative		Positive	Negative		Positive	Negative	
Age (years)	≤ 20	33	9	24	0.550	17	16	0.830	21	12	0.1482
	21–30	12	5	7		7	5		8	4	
	≥ 31	13	3	10		6	7		12	1	
Gender	Male	34	7	27	0.082	16	18	0.397	22	12	0.233
	Female	24	10	14		14	10		19	5	
Tumor location	Limbs	51	16	35	0.352	27	24	0.617	35	16	0.352
	Others	7	1	6		3	4		6	1	
Stage	IIA	14	6	8	0.335	7	7	0.226	9	5	0.816
	IIB	32	9	23		18	14		23	9	
	III	12	2	10		5	7		9	3	
Lung metastasis	Yes	20	3	17	0.082	10	10	0.849	15	5	0.601
	No	38	14	24		20	18		26	12	
Local recurrence	Yes	22	3	19	0.040^*^	11	11	0.837	18	4	0.146
	No	36	14	22		19	17		23	13	
Histological type	Osteoblastic	34	13	21	0.238	21	13	0.338	26	8	0.352
	Fibroblastic	3	0	3		1	2		3	0	
	Chondroblastic	18	4	14		7	11		10	8	
	Others	3	0	3		1	2		2	1	
Tumor size	< 8 cm	41	13	28	0.533	24	17	0.107	28	13	0.533
	≥ 8 cm	17	4	13		6	11		13	4	

^**#**^Pearson’s χ^2^ test

^*^With significant difference (*P* < 0.05)

The details of the human benign bone and soft tissue tumor specimens are show in Additional file 1.

### Cell culture

The human osteosarcoma cell line SaOS2 was obtained from Cellcook Biological Technology Co., Ltd. (Guangzhou China). The malignant transformed hFOB1.19 cell line (MTF cells) was produced in our laboratory, as previously reported [7]. All the cells were maintained in high-glucose Dulbecco’s modified Eagle’s medium (DMEM, Hyclone, Logan, UT, USA) supplemented with 10% fetal bovine serum (FBS, BI, Kibbutz Beit Haemek, Israel) and 1% penicillin-streptomycin (Hyclone). All the cells were cultured at 37 °C in 5% CO2 and humidified atmosphere.

Cells were treated with pYEEI (100 mM, Src activator, Enzo Life Science, The Netherlands), BEZ235 (500 nM, PI3K inhibitor, MedChem Express, USA) or IGF-1 (100 ng/ml, Akt activator, R&D System, USA), chloroquine (CQ, 8 μM autophagy flux inhibitors, Sigma-Aldrich, USA) for 12 h, separately or in combination.

### Transfection

The pLVX-mCMV-ZsGreen expressing lentiviral vector for TSSC3 and the control was synthesized and obtained from Chongqing Maobai Technology Co., Ltd. (Maobai, Chongqing, China) and the coding sequence of *TSSC3* was amplified using the primers 5′-CCGGAATTCGCCACCATGAAATCCCCCGACGAGGTGCTAC-3′ and 5′-CGCGGATCCTCACTTATCGTCGTCATCCTTGTA-3′. The short hairpin RNA (shRNA) in the pHBLV-U6-Puro lentiviral vector targeting *ATG5* and its control (scrambled) were purchased from Hanbio Biotechnology Co., Ltd. (Hanbio, Shanghai, China). MTF and SaOS2 cells were infected with the lentivirus vectors according to the manufacturer’s instructions and as previously described [9, 10]. The shRNA target sequences are listed in Additional file 2: Table S1). The overexpression and knockdown function was verified using quantitative real-time PCR and western blotting analysis.

### Cell proliferation, cell viability, and colony formation assays

For the cell proliferation assay, a cell light 5-ethynyl-2′-deoxyuridine (EdU) imaging kit (C0075S, Beyotime, Shanghai, China) was used according to the manufacturer’s instructions (Edu; 10 μM, 2 h, for MTF cells and 20 μM, 2 h, for SaOS2 cells). Cells were observed under an inverted phase contrast fluorescence microscope (Olympus, Tokyo, Japan). Cell counting kit-8 (CCK8, C0038, Beyotime) and colony formation assays were carried as described previously [8, 30].

### Transmission electronic microscopy

The transmission electron microscopy (TEM) assay was performed as previously described [8]. Briefly, cells (1 × 10^6^) were harvested after transfection with lentivirus overexpressing TSSC3 (overTSSC3) and its control (overCtrl), while fresh tissues were dissected, fixed with 4% glutaraldehyde in phosphate-buffered saline (PBS) at 4 °C overnight, post-fixed in 1% osmium tetroxide, dehydrated with ethanol, embedded in Epon, and then stained with aqueous uranyl acetate and lead citrate before being observed. Images were acquired using a HT7700 electron microscope (HITACHI, Tokyo, Japan). The number of autophagic vacuoles (AVs), including autophagosomes and autolysosomes, in each cell was quantified in 20 randomly selected cells of each group [31].

### Quantitative real-time PCR analysis and western blot analysis

Quantitative real-time PCR (qPCR) and western blotting analyses were performed as previously described [32]. The primers used in this study are listed in Additional file 2: Table S2 and the details of western blotting are listed in Additional file 1.

### Immunofluorescence, apoptotic analysis, histology, and immunohistochemistry

The assays were performed as previously described [11, 32]. The details are shown in Additional file 1.

### Wound healing and Transwell assays

Wound healing and Transwell assays were performed as previously described [4, 11]. Briefly, after appropriate treatments, cells were seeded in 6-well plates and cultured until they reached 90% confluence. A 10-μl micropipette tip was used to make a wound. Cells were monitored at 0 h and 48 h after scratching and images of wound healing were captured (magnification of 100×) using a inverted phase contrast light microscope (Olympus, Tokyo, Japan) with DP Controller software (Olympus Life Science, Tokyo, Japan). Cell migration was quantified by measuring the wound healing index; i.e., the wound area healed by the cells at 48 h after scratching relative to the wound area at 0 h, using ImageJ software. For the Transwell migration or invasion assays, cells were resuspended in DMEM without serum and seeded into the upper chamber of 8-μm Transwell filters (Merck Millipore, Berlin, Germany). The invasion assay was performed using filters pre-coated in 1:3 diluted matrigel (BD Biosciences, Bedford, MA, USA) while the migration assay was not. DMEM containing 15% FBS was added to the lower chambers (24-well plate) and the cells were incubated 16 h for the migration assay and 24 h for the invasion assay. The invaded or migrated cells were quantified after 0.1% crystal violet staining in five randomly selected fields (magnification of 200×).

### Xenografts

Xenograft models were generated in 6-week-old female nude mice (Laboratory Animal Center, Xinqiao Hospital, TMMU). Eighteen Mice were randomly divided into three groups (six for overCtrl and scrambled MTF cells; six for overTSSC3 and scrambled MTF cells; and six for overTSSC3 and shATG5 MTF cells) and weighed every four days. Cells suspensions of 4 × 10^6^ cells/ml in PBS were injected subcutaneously into a single side of the infra-axillary of each mouse in a volume of 0.1 ml. Xenografts were observed and measured every three days. The volume of xenografts were calculated as V (mm^3^) =1/2 × (length × width^2^) [33]. Mice were sacrificed at 20 days after injection and tumors were harvested and measured. A small part of tumors were excised and fixed with 4% glutaraldehyde quickly on ice for TEM. The remainder was fixed with 10% neutral buffered formalin, sliced, subjected to hematoxylin and eosin (H&E) staining, and further analyzed by immunohistochemistry (IHC).

### In vivo lung metastasis model

Twenty-one nude mice were randomly divided into three groups as mentioned above. Cell suspensions of 5 × 10^7^ cells/ml were injected into the tail vein of 4-week-old female nude mice (Laboratory Animal Center, Xinqiao Hospital, TMMU) in a volume of 100 μl. The mice were evaluated every two days for weight and emaciation incapacitating tumor burden. Natural deaths of the nude mice were recorded to calculate the lifetime at 38 days after injection. The surviving nude mice were sacrificed at 38 days. All the lungs were resected and fixed in 10% neutral buffered formalin, and the fixed lungs were embedded in paraffin, sectioned, and stained with H&E. Microscopic lung metastases were counted under a light microscope after H&E staining. Five discontinuous and deep sections were used to define whether there was a metastasis nodule in the fixed lungs and the number of lung metastasis nodules was counted in one section with the most nodules.

All the animal care and experimental procedures were approved by the Institutional Animal Care and Use Committee of Xinqiao Hospital, TMMU, and were performed according to the Guide for the Care Use of Laboratory Animals.

### Statistical analysis

Quantitative data are presented as the mean ± SD. Quantitative data were analyzed using unpaired Student’s *t*-tests for two groups and analysis of variance (ANOVA) with Bonferroni’s multiple comparisons for three or more groups. Categorical data were analyzed using the Chi-squared test or Fisher’s exact test, and Spearman rank correlation coefficients. Survival analysis was carried out using the Kaplan–Meier method with the log-rank test. The independent prognostic factors were determined by Cox regression analysis. *P* < 0.05 was considered statistically significant. All analyses were performed using SPSS 20.0 software (version 20.0, SPSS Inc., Chicago, IL, USA) or GraphPad Prism (version 7.00, GraphPad Software, Inc., San Diego, CA, USA). In Vitro experiments were performed at least triplicate.

## Results

### ATG5 expression correlates positively with TSSC3 expression in osteosarcoma tissues

**Fig. 1:**
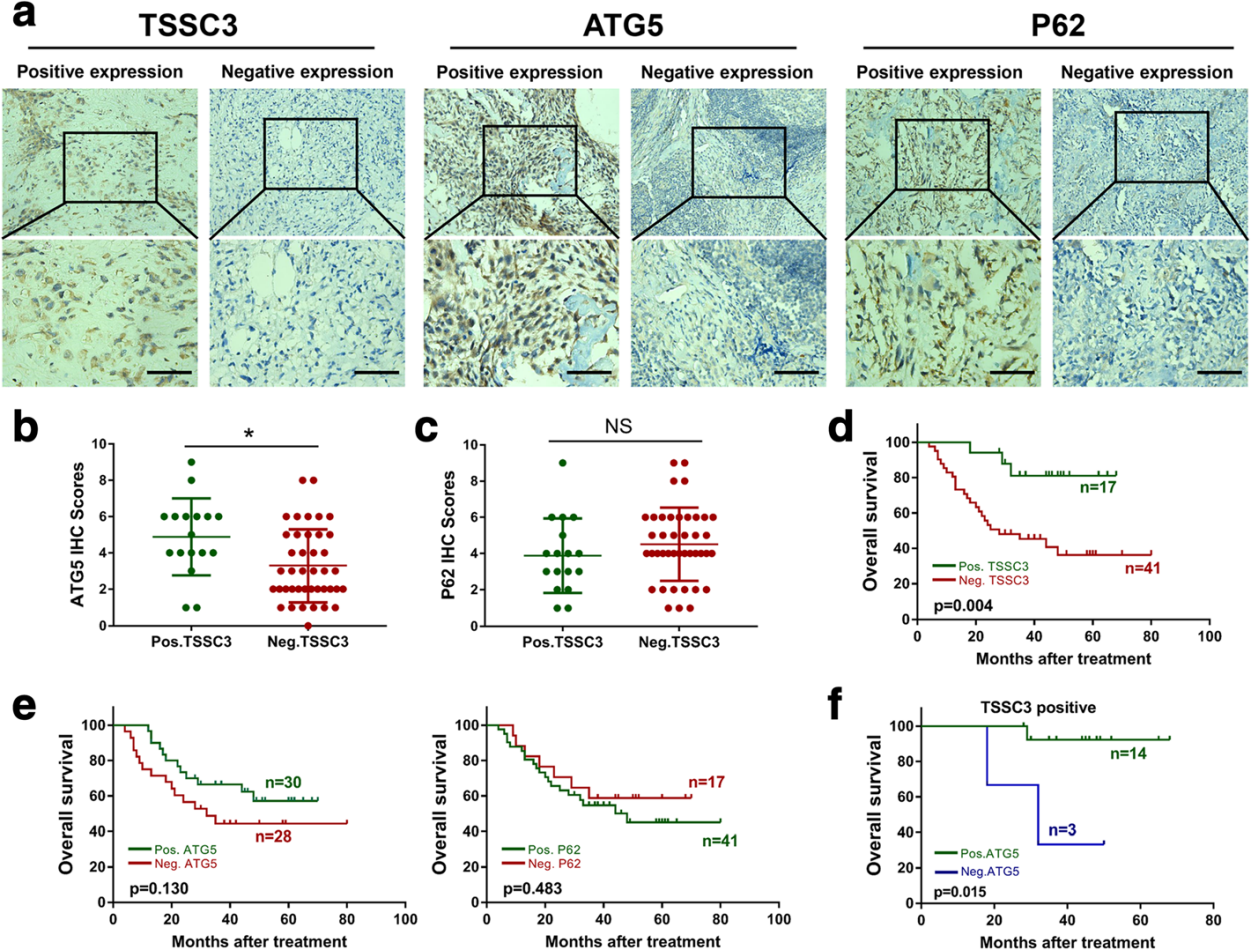
ATG5 expression correlated positively with TSSC3 expression in osteosarcoma and predicts a favorable prognosis. **a** Representative images (200× and 400×) of immunohistochemistry (IHC) positive or negative staining for TSSC3, ATG5, and P62 in human osteosarcoma tissues. A mass of yellow or brown color in tumor cells is considered as a positive staining. Scale bars: 50 μm. **b** Higher IHC scores of ATG5, which symbolized protein expression intensity, was found in patients with TSSC3(+) osteosarcoma compared with that in patients with TSSC3(−) osteosarcoma. **c **There was no significant correlation between TSSC3 and P62. *P < 0.05. **d** Kaplan–Meier curves showing that positive expression of TSSC3 is significantly associated with an improved prognosis and longer overall survival (P < 0.05), **e** while ATG5 and P62 expression is not (*P* > 0.05). **f** Kaplan–Meier curve showing that positive expression for both TSSC3 and ATG5 is significantly associated with a favorable prognosis (*P* < 0.05)

Previously, we proved that *TSSC3* mRNA expression is decreased in human osteosarcoma tissues compared with that in non-tumor tissues [8]. To examine the correlation between TSSC3 and autophagy, we first applied immunohistochemical staining to examine the TSSC3, ATG5, and P62 expression in human benign bone and soft tissue tumors, and osteosarcoma. We observed higher rate of TSSC3 and ATG5 positivity in fibrous dysplasia and osteoblastoma compared with that in osteosarcoma (*P* < 0.05), while the positive rate of P62 expression was lower but not statistically significant (*P* > 0.05) in fibrous dysplasia and osteoblastoma compared with that in osteosarcoma (Additional file 3: Figure S1 a-c). The results showed that TSSC3 and autophagy-related proteins expression were in good agreement with various tendencies of benign bone and soft tissue tumors and osteosarcoma suggesting a potential correlation between them in osteosarcoma. To further confirm this, 58 human osteosarcoma tissues were used to investigate the relevance of TSSC3, ATG5, and P62 expression in vivo. The representative images of positive or negative IHC staining of TSSC3, ATG5, and P62 are shown in Fig. 1a. Interestingly, a higher IHC score for ATG5 was found in osteosarcoma patients with positive expression of TSSC3 (Fig. 1b). Further analysis demonstrated that the TSSC3 expression is positively correlated with that of ATG5. (Additional file 2: Table S3). However, we failed to detect a significant correlation between TSSC3 and P62 expression (Fig. 1c and Additional file 2: Table S3). Overall, these data suggested that ATG5 expression positively correlated with TSSC3 expression in osteosarcoma tissues.

### Positive expression of ATG5, associated with positive TSSC3, predicts a favorable prognosis of osteosarcoma

We then used IHC staining of above tissues to investigate the correlations between TSSC3, ATG5, and P62 expression and clinicopathological features of osteosarcoma. Among the 58 patients, there were 34 males and 24 females. The median age was 22 years old (range: 8–59 years old). The follow-up time ranged from 4 (death) to 80 months. Twenty-two patients had local recurrence after chemotherapy and surgery of the primary osteosarcoma, 12 patients had lung metastasis at diagnosis, and eight more patients developed lung metastasis after the treatment (Table 1). Moreover, we found that expression of TSSC3 was significantly associated with a lower local recurrence rate (*P* < 0.05), but not with age, sex, stage, location, size, and pathological subtype. The rate of TSSC3 positivity was 36.8% (14/38) in osteosarcoma without metastasis and 15.0% (3/20) in osteosarcoma with metastasis; however, the decrease was not significant (*P* = 0.082) (Table 1). Surprisingly, neither the expression of ATG5 nor P62 seemed to be associated with the clinicopathological features of osteosarcoma (Table 1). As expected, Kaplan-Meier curves showed positive expression of TSSC3 was significantly related to an improved OS (Fig. 1d), while the differential expression of ATG5 and P62 had no notable impact on OS (Fig. 1e). Furthermore, TSSC3 protein expression, tumor size, and metastasis were validated to be independent prognostic markers for OS of osteosarcoma by univariate and multivariate Cox regression (Additional file 2: Table S4 and Table 2). We further observed that positive expression of TSSC3 and ATG5 was significantly associated with an earlier Enneking stage and there were markedly lower metastasis and recurrence rate (7.1 and 14.3%) for positive ATG5 expression compared to negative ATG5 expression (66.7 and 33.3%) in TSSC3(+) osteosarcoma; however, these differences were not statistically significant (Additional file 2: Table S5). Thus, we inferred that positive ATG5 expression in patients with TSSC3(+) osteosarcoma might suggest a favorable prognosis. To test this hypothesis, Kaplan–Meier analysis showed the patients with positive expression of both TSSC3 and ATG5 displayed a more favorable OS (Fig. 1f). Taken together, these results showed that TSSC3 is an independent prognostic marker for OS and positive expression of both TSSC3 and ATG5 is a potential predictor of favorable prognosis in osteosarcoma.

**Table 2:** Multivariate Cox proportional hazard regression analysis for overall survival in patients with osteosarcoma

Variable	No.	Hazard ratio (95% Confidence interval)	P
Tumor size, cm, ≥ 8 cm (vs. < 8 cm)	17/58	3.657 (1.592 to 8.404)	0.002^*^
Lung metastasis (yes vs. no)	20/58	7.053 (2.975 to 16.723)	< 0.001^*^
TSSC3 negative expression (vs. positive expression)	41/58	3.795 (1.126 to 12.785)	0.031^*^

^*^With significant difference (*P* < 0.05)

### TSSC3 overexpression enhances autophagic flux in osteosarcoma cells in vitro

**Fig. 2:**
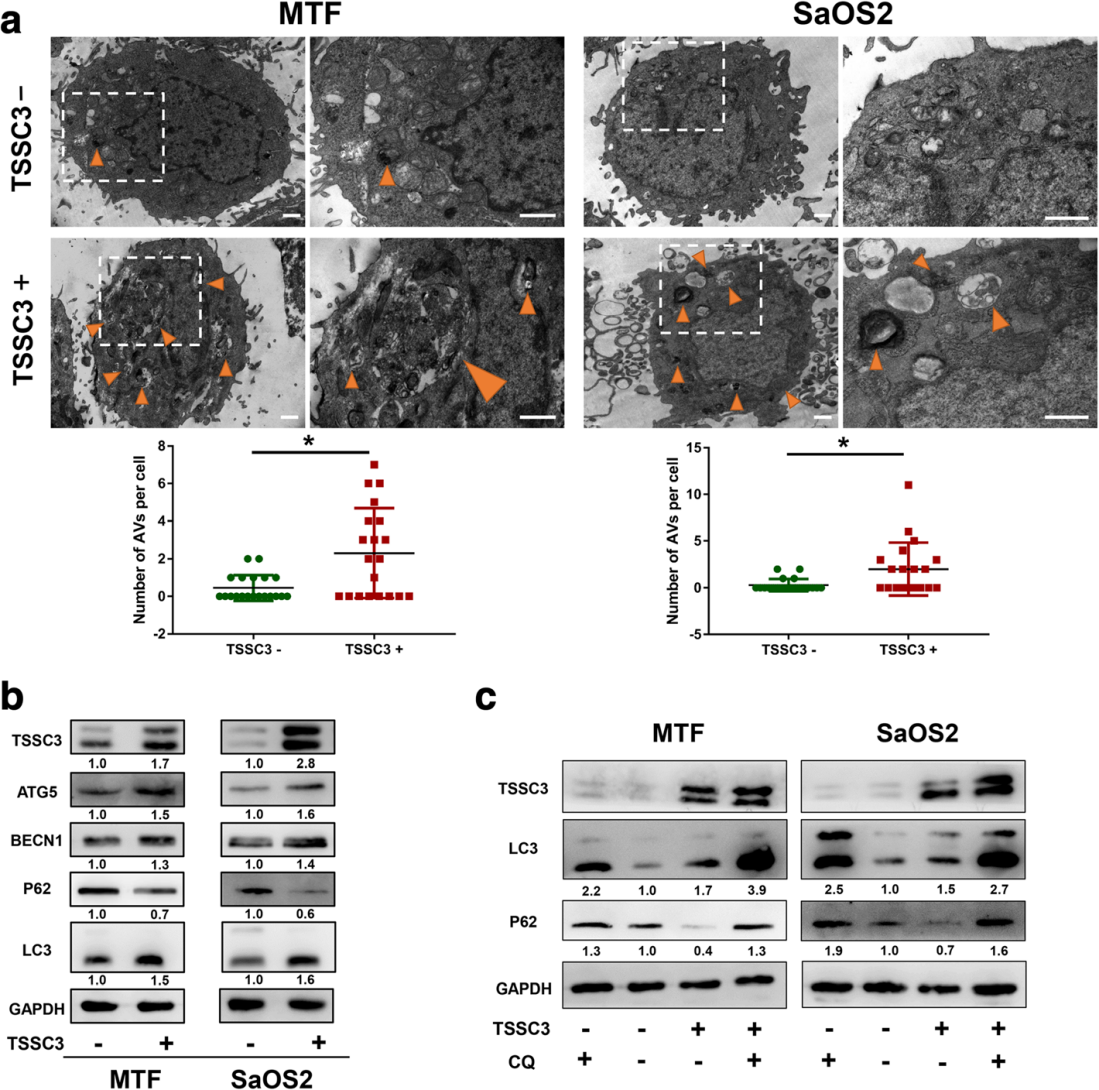
TSSC3 overexpression enhances autophagy flux in osteosarcoma cells in vitro. **a** MTF and SaOS2 cells were stably transfected as indicated. Representative electron microscopic images showing that an increased number of autophagic vacuoles (AVs; autophagosomes and autolysosomes) are present in MTF and SaoS2 cells transfected with lenti-overTSSC3. The numbers of AVs in each cell were quantified by randomly selecting 20 cells per group. *P < 0.05, Scale bars: 1 μm. **b** Representative images of the western blotting analysis of autophagy-related proteins levels in MTF and SaOS2 cells. Cells were treated with or without TSSC3-overexpression. Protein levels on the western blots were quantified by densitometry of TSSC3, ATG5, BECN1, LC3-II, and P62, normalized to GAPDH and shown as a fold change compared with that of the control (TSSC3-) groups. Quantification data was in Additional file 3: Figure S2d. **c** Western blotting of LC3-II and p62 in cells following TSSC3-overexpression or treatment with autophagy flux inhibitors CQ for 12 h as indicated. Protein levels on the western blots were quantified by densitometry of LC3-II and P62, normalized to GAPDH, and shown as a fold change compared with that of the control (TSSC3- and CQ-) groups

We found ATG5 expression is positively correlated with TSSC3 expression in osteosarcoma samples, and the relationship between TSSC3 and autophagy had never been reported before. Therefore, we examine the effect of TSSC3 in regulating autophagy in osteosarcoma cells. First, we stably overexpressed TSSC3 using lenti-overTSSC3 in MTF and SaOS2 cells and examined the formation of AVs by using TEM. The results demonstrated that there was an increased number of large diameter autophagosomes and autolysosomes in MTF or SaOS2 cells transfected with lenti-overTSSC3 transfected (Fig. 2a). Consistent with this, western blotting analysis revealed an accumulation of ATG5, BECN1, and lipid-bound LC3-II in TSSC3-overexpressing cells (Fig. 2b), which indicated an increase in the synthesis of autophagosomes [23]. In complementary experiments, immunofluorescence assays demonstrated that BECN1, ATG5, and LC3B levels were significantly increase in TSSC3-overexpressing cells (Additional file 3: Figure S2 a). In addition, overexpression of TSSC3 in osteosarcoma cell lines increased *BECN1* and *ATG5* mRNA levels (Additional file 3: Figure S2b). Furthermore, to investigate the effect of TSSC3 on autophagic flux, the expression of P62, also known as sequestosome 1 (SQSTM1), a substrate of autophagy [34], was examined. As expected, TSSC3 overexpression decreased P62 protein levels (Fig. 2b), suggesting enhanced autophagic flux. To verify this result, the lysosomal autophagy inhibitor CQ was used, which resulted in increased LC3-II and P62 accumulation in TSSC3-overexpressing cells (Fig. 2c), supporting the notion that TSSC3 overexpression did not block autophagic flux. Taken together, these data suggested that TSSC3 overexpression induced autophagy and enhanced autophagic flux in osteosarcoma cells in vitro.

### TSSC3-mediated autophagy contributes to TSSC3-induced inhibition of malignant proliferation of osteosarcoma cells

**Fig. 3:**
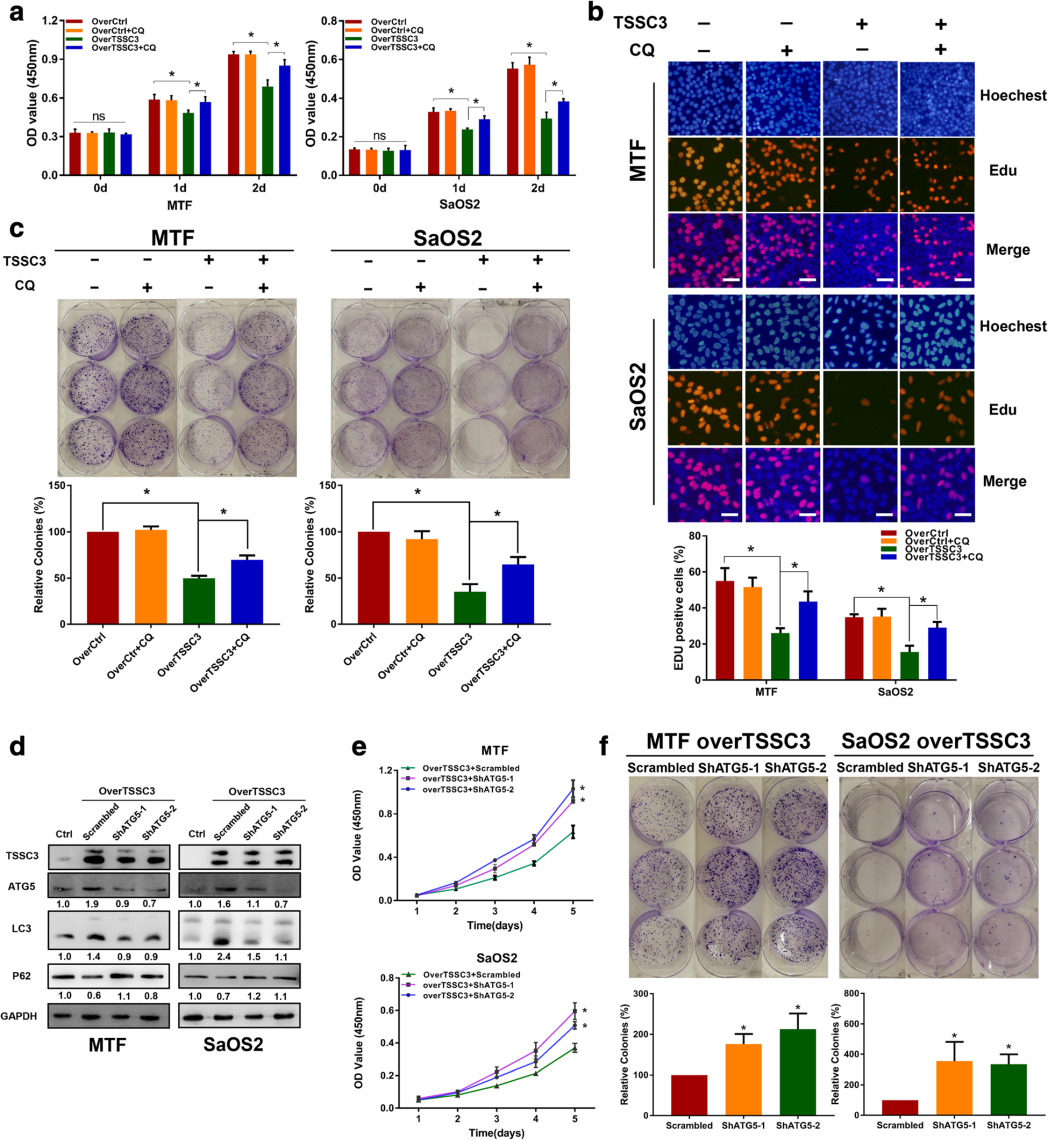
TSSC3-mediated autophagy contributes to TSSC3-induced inhibition of malignant proliferation of osteosarcoma cells. **a** Blocking autophagy flux using CQ (8 μM, 24 h) attenuates TSSC3-induced inhibition of cell viability, as determined by a CCK-8 assay, as indicated for 2 days. Cells were plated in 96-well plates at 5 × 10^3^ cells for each well. *P < 0.05, NS: No Significance. **b** The number of EdU positive cells decreased significantly after transfection with the TSSC3-overexpression plasmid in MTF and SaOS2 cells, but partly recovered after combined treatment with CQ (8 μM, 12 h). *P < 0.05, Scale bars: 50 μm. **c** Blocking autophagy flux with CQ (5 μM, 4 days) attenuates TSSC3-induced inhibition of colony-formation ability. Cells were plated in 6-well plates at 200 cells per well for MTF cells and 400 cells per well for SaOS2 cells. Photographs of colony formation were taken after 11 days of culture for MTF cells or 12 days of culture for SaOS2 cells. *P < 0.05. **d** Western blotting analysis showing that ATG5 knockdown blocked TSSC3-induced autophagy. Protein levels on the western blots were quantified by densitometry of ATG5, LC3-II, and P62 are normalized to GAPDH and shown as the fold change relative to the Ctrl groups. Quantification data was in Additional file 3: Figure S2e. **e** ATG5 knockdown blocked TSSC3-induced inhibition of cell proliferation. The viability of MTF-TSSC3-Scrambled/shATG5–1 or 2 and SaOS2-TSSC3-Scrambled/shATG5–1 or 2 cells was measured using CCK-8 assays. Cells were plated in 96-well plates at 1 × 10^3^ cells per well. *P < 0.05, compared with the TSSC3-Scrambled group. **f** Photographs of colony formation for the cells mentioned in (**e**), which were plated in 6-well plates at 200 cells per well and 12 days for MTF cells, or 16 days for SaOS2 cells after plating. These quantitative data show that ATG5 knockdown blocked TSSC3-induced inhibition of the colony-formation ability of osteosarcoma cells. *P < 0.05

Autophagy has been reported as a tumorigenesis suppressor in some situations [35, 36] and we have found TSSC3 overexpression enhanced autophagy flux and inhibited cell proliferation (Additional file 3: Figure S3 a) in osteosarcoma cells [8]. Thus, to determine whether TSSC3-induced inhibition of osteosarcoma malignant proliferation was related to autophagy, we applied the CCK-8 assay to detect the cell viability with or without TSSC3-overexpression, with or without autophagy suppression by CQ. We found that overexpression of TSSC3 significantly decreased the cell viability of MTF and SaOS2 cells, and no obvious cytotoxicity was found compared with the control group when the cells were treated with CQ alone. By contrast, CQ significantly blocked the autophagic flux (Additional file 3: Figure S2 c), and cell viability was partly rescued in TSSC3-overexpressed cells when treated with CQ (Fig. 3a). Subsequently, we found that blocking autophagy with CQ attenuated the TSSC3-induced suppression of proliferation and colony-formation ability of MTF and SaOS2 cells, as assessed using EdU and colony formation assays (Fig. 3b and c). These data indicated that TSSC3-induced inhibition of malignant proliferation of osteosarcoma cell lines might be related to autophagy. Moreover, to further confirm this notion, enhanced autophagy was eliminated via *ATG5* knockdown using shRNA lentiviruses (two effective sequences were chosen), which were stably transfected into TSSC3-overexpressing cells; western blotting analysis confirmed this result (Fig. 3d). We also found that blocking autophagy (ATG5 knockdown) reduced TSSC3-induced inhibition of cell proliferation and colony-formation ability in MTF and SaOS2 cells (Fig. 3e and f). Accumulating evidence shows that autophagy can promote apoptosis to suppress tumor progression [37]. Therefore, to determine whether TSSC3-mediated autophagy could suppress malignant proliferation depending on apoptosis in osteosarcoma cell lines, Hoechst and Annexin V-PE/7-AAD staining were used. As shown in Additional file 3: Figure S3 b and c, inhibition of autophagy by CQ partly abrogated TSSC3-induced apoptosis in SaOS2 cells, which was consistent with the markedly upregulation of cleaved caspase-3 and Bax, and reduced Bcl2 (Additional file 3: Figure S3 d). Intriguingly, autophagy inhibition had no visible effect of TSSC3-induced apoptosis in the MTF cell line (Additional file 3: Figure S3 b-d). These differences may be derived from the genetic background of the cell lines. Collectively, these results suggested that TSSC3-mediated autophagy contributes to the anti-osteosarcoma effect of TSSC3 by suppressing cell malignant proliferation, which may not completely depend on autophagy-triggered apoptosis.

### Crucial involvement of autophagy in TSSC3-mediated tumorigenesis in osteosarcoma cells in vivo

**Fig. 4:**
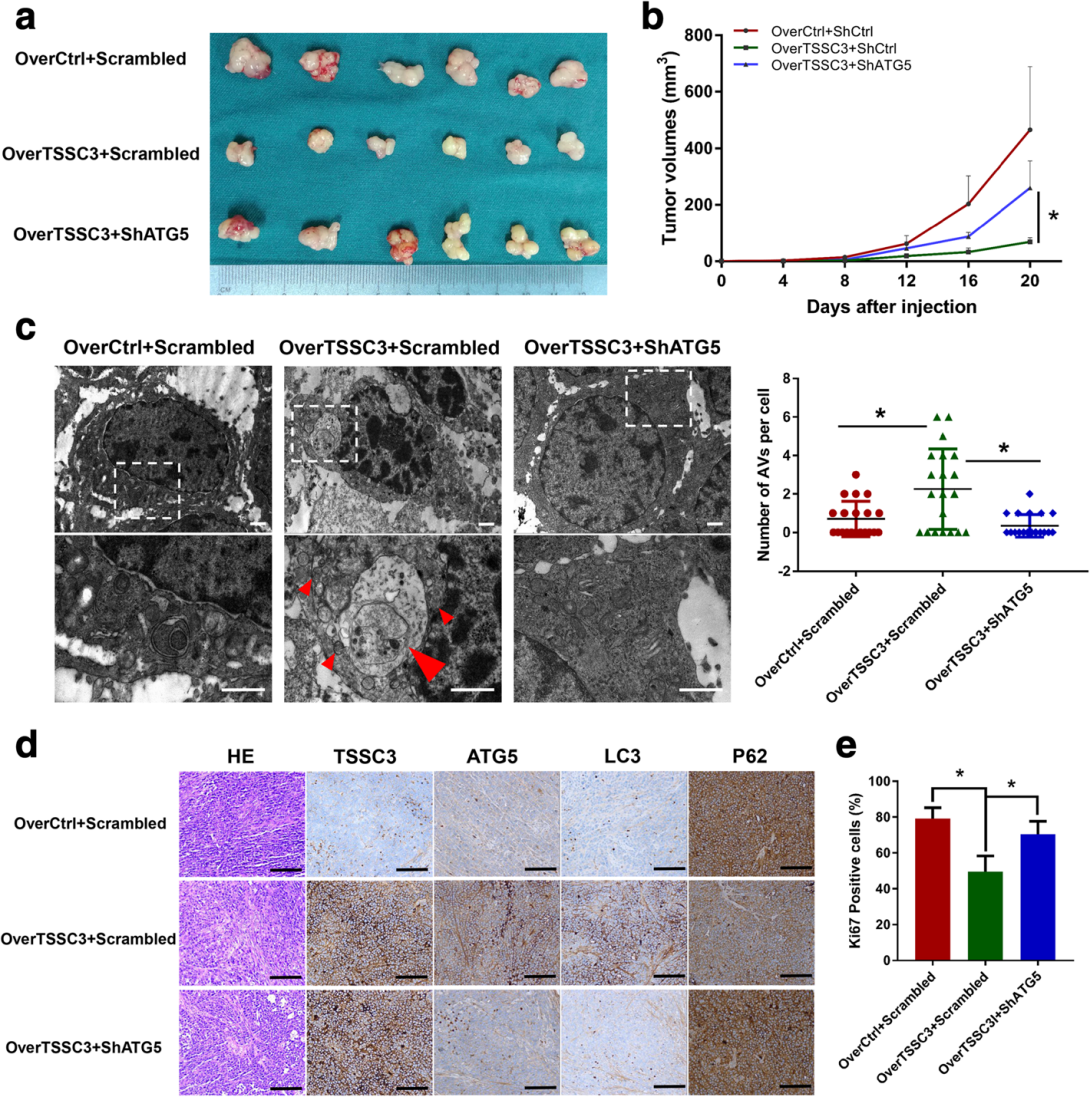
Crucial involvement of autophagy in TSSC3-mediated tumorigenesis in osteosarcoma cells in vivo. **a** Representative xenograft tumors of sacrificed nude mice at the end of the experiment with treatments as indicated. **b** Growth curves of subcutaneous xenograft tumors show that lenti-overTSSC3 transfected MTF cells displayed a reduced ability to form tumors in nude mice, while their combination with lenti-shATG5 blocked the ability of TSSC3 to inhibit the tumorigenesis of osteosarcoma in vivo. *P < 0.05. **c** Representative electron microscopy images showing that TSSC3-overexpression induces autophagy and ATG5 knockdown blocked TSSC3-induced autophagy in vivo. The numbers of autophagic vesicles (AVs) in each cell was quantified in 20 randomly selected cells per group. * P < 0.05, Scale bars: 1 μm. **d** Confirmation of the effect of TSSC3-overexpression and ATG5 knockdown on autophagy in osteosarcoma cells in vivo using autophagy-related proteins immunohistochemical staining. The quantitation of IHC scores is shown in Additional file 2: Figure S4). Scale bars: 100 μm. **e** Quantification of Ki67 staining in xenografts from MTF cells treated as indicated. Representative images are shown in the Additional file 2: Figure S4). *P < 0.05

Having shown that TSSC3 mediates autophagy and induces inhibition of malignant proliferation of osteosarcoma cells in vitro, it was important to determine whether these effects could be detected in vivo. We established subcutaneous xenograft models by stably transfecting MTF cells into athymic nude mice. Then, we found that TSSC3 overexpression significantly reduced tumorigenesis, as reflected by the tumor size, volume, body weight, and tumor weight of the xenograft tumors. However, knockdown of *ATG5* restored tumorigenesis in TSSC3-overexpressing cells (Additional file 3: Figure S4 a-b and Fig. 4a-b). Moreover, that overexpression of TSSC3 enhanced autophagy and knockdown of *ATG5* blocked TSSC3-induced autophagy were further confirmed in vivo using TEM (Fig. 4c) and IHC staining of TSSC3 and autophagy related proteins of human xenograft tumor tissues (Fig. 4d and Additional file 3: Figure S4c). As expected, the rate of positive Ki67 IHC staining was high in the control groups, decreased in the TSSC3-overexpressing group, and restored in the TSSC3-overexpressing and ATG5-knockdown groups, suggesting that autophagy contributes to TSSC3-induced inhibition of malignant proliferation in vivo (Additional file 3: Figure S4d and Fig. 4e). Thus, all of the *vitro* and in vivo results indicated that TSSC3-induced autophagy plays a crucial role in TSSC3-mediated tumorigenesis in osteosarcoma cells.

### TSSC3 inhibits osteosarcoma cells migration and invasion associated with autophagy

**Fig. 5:**
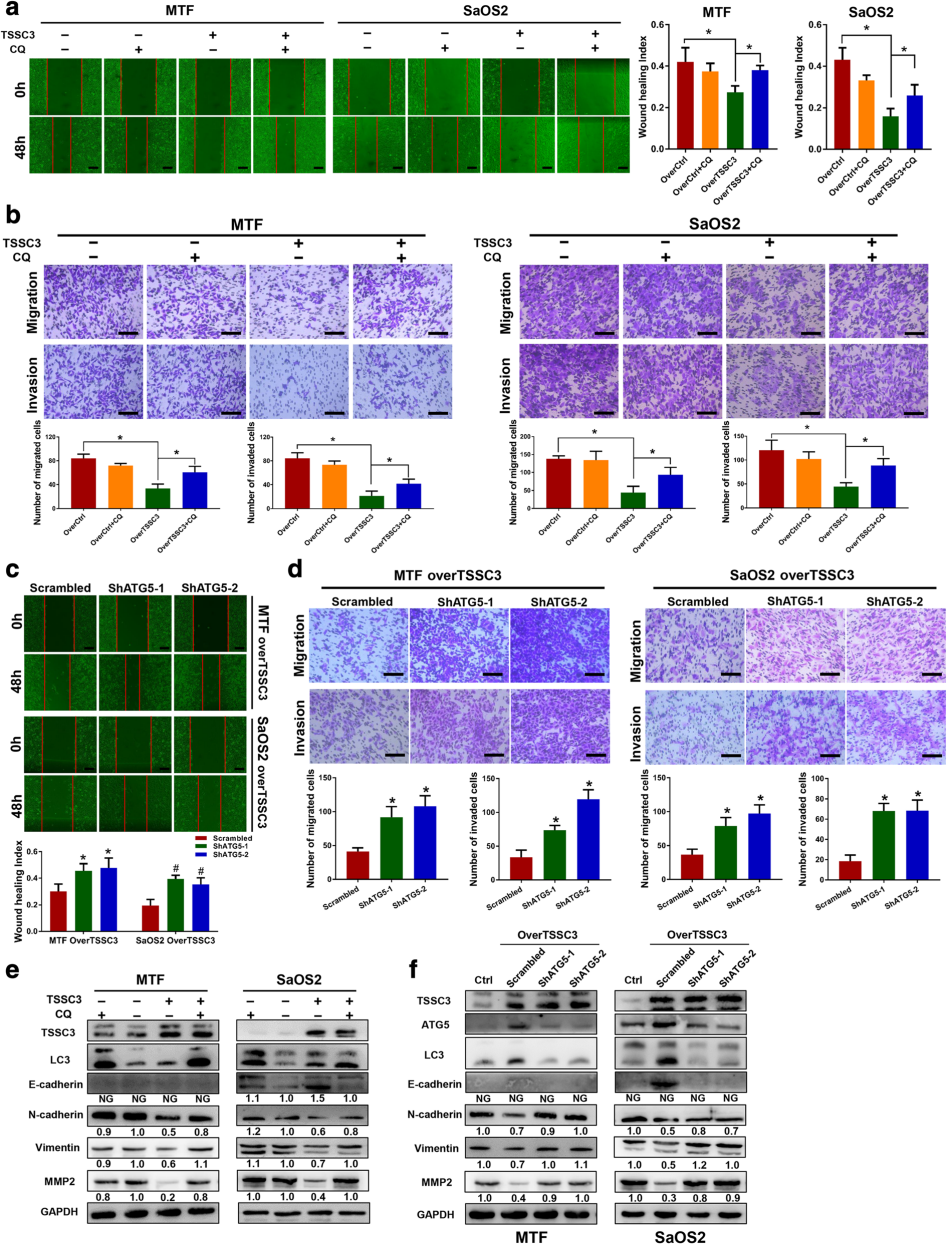
TSSC3 inhibits osteosarcoma cell migration and invasion associated with autophagy. **a** A wound healing assay was conducted for over-Ctrl and TSSC3-overexpression MTF or SaOS2 cells treated with or without CQ (8 μM, 12 h) for 48 h. * *P* < 0.05, Scale bars: 200 μm. **b** Transwell migration and invasion assays indicate blockage of the autophagy flux by CQ (8 μM) alleviates TSSC3-induced inhibition of osteosarcoma cells migration and invasion in vitro. * *P* < 0.05, Scale bars: 200 μm. **c** ATG5 knockdown blocked TSSC3-induced suppression of cell migration. MTF-TSSC3-Scrambled/shATG5–1 or 2 and SaOS2-TSSC3-Scrambled/shATG5–1 or 2 cells migration abilities were measured using a wound healing assay for 48 h. * *P* < 0.05, compared with the MTF-Scrambled group, ^#^ *P* < 0.05, compared with the SaOS2-Scrambled group, Scale bars: 200 μm. **d** Transwell migration and invasion assays with cells treated as mentioned above also show that ATG5 knockdown blocked TSSC3-induced suppression of cell migration. * *P* < 0.05, Scale bars: 200 μm. **e-f** Representative images of western blotting analysis of autophagy-related proteins and epithelial-mesenchymal transition and invasion-related proteins levels in MTF and SaOS2 cells. Cells were treated with or without TSSC3-overexpression and TSSC3-induced autophagy was blocked using CQ or shATG5. Protein levels on the western blots were quantified by densitometry of E-cadherin, N-cadherin, Vimentin and MMP2, normalized to GAPDH and given as the fold change compared with that in the control groups. NG: Not given. Quantification data was in Additional file 3: Figure S5

Metastasis is a major factor predicting poor prognosis in patients with osteosarcoma [3] and autophagy depends on an environment that may limit the invasion and dissemination of tumor cells from a primary site [21, 36]. We found that positive expression of both TSSC3 and ATG5 is significantly associated with an improved prognosis and correlated negatively with metastasis in human osteosarcoma; therefore, we speculated that TSSC3-induced autophagy might have an effect on the regulation of osteosarcoma cell metastasis. To confirm this speculation, we used wound healing and Transwell assays. The results showed that TSSC3 overexpression significantly decreased the motility and invasive ability of MTF and SaOS2 cells, but had no significant effect when the cells were treated with CQ alone, compared with that in the control group. Although the inhibitory effects of TSSC3 overexpression on cell motility and invasive ability were markedly attenuated by CQ treatment (Fig. 5a and b), knockdown of I in TSSC3-overexpressing MTF and SaOS2 cells significantly improved cell motility and invasion (Fig. 5c and d). Thus, these results confirmed the findings that TSSC3 overexpression-induced autophagy suppresses osteosarcoma cell metastasis in vitro. To further investigate the mechanism, we observed that the epithelial cell marker E-cadherin was upregulated in TSSC3-overexpressing SaOS2 cells (but not in MTF cells), whereas the expression levels of mesenchymal markers N-cadherin, Vimentin, and MMP2 were inhibited. These protein expression levels did not increase significantly when the cells were treated with CQ alone to block autophagy. However, interestingly, the N-cadherin, Vimentin, and MMP2 levels increased markedly when TSSC3-overexpressing MTF and SaOS2 cells were treated with CQ (a decrease in the E-cadherin level was also detected in TSSC-overexpressing SaOS2 cells) (Fig. 5e and f). The epithelial-mesenchymal transition (EMT) is a metastatic behavior of cancer cells [38]. Thus, the impairment of MTF and SaOS2 cell migration and invasion might be caused by inhibition of the EMT, dependent on TSSC3 overexpression-induced autophagy.

### Autophagy contributes to TSSC3-induced lung metastasis suppression in vivo

**Fig. 6:**
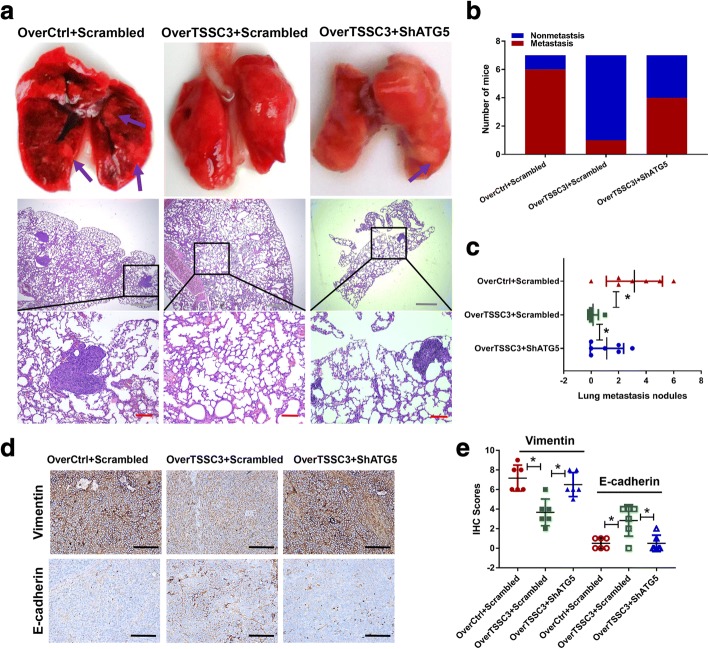
Autophagy contributes to TSSC3-induced lung metastasis suppression in vivo. MTF cells were transfected as indicated and injected into the tail vein of nude mice to establish an in vivo model of lung metastasis. **a** Representative macroscopic and microscopic images (H&E staining) of the lungs. Purple arrows indicate possible metastatic lesions. Gray scale bars: 500 μm, red scale bars: 100 μm. **b** Percentage of mice bearing lung metastases in each group (*n* = 7). **c** Numbers of lung metastatic nodules quantified on the section with H&E staining of the lungs. *P < 0.05. **d-e** E-cadherin and Vimentin protein expression in xenograft tumors, quantified by immunohistochemistry (IHC). The quantification of the IHC scores is shown in **e**. *P < 0.05

To further investigate whether autophagy contributes to TSSC3-induced suppression of metastasis in osteosarcoma, we established a lung metastasis model in vivo by injecting stably transfected MTF cells into the tail vein of athymic nude mice. We found that overexpression of TSSC3 notably reduced the ability of MTF cells to induce lung metastases, as reflected by the decreased incidence and number of metastatic nodules and the increased survival time of the mice. However, knockdown of *ATG5* restored the ability of TSSC3-overexpressing cells to establish lung metastases (Fig. 6a-c and Additional file 3: Figure S6b). In addition, using IHC staining, the metastatic nodules were negative for E-cadherin and CK18, but positive for Vimentin, indicative of a mesenchymal origin (Additional file 3: Figure S6a). Consistent with these findings, the expression change of E-cadherin and Vimentin in subcutaneous xenograft tumors induced using transfected cells were measured using IHC staining (Fig. 6d and e), which indicated that TSSC3-induced autophagy blocked the EMT of osteosarcoma cells in vivo. These results, together with our in vitro findings, highlighted a functional role for autophagy in the regulation of TSSC3-induced lung metastasis suppression in osteosarcoma.

### TSSC3 overexpression induces autophagy in osteosarcoma cells via inactivating the Src-mediated PI3K/Akt/mTOR pathway

**Fig. 7:**
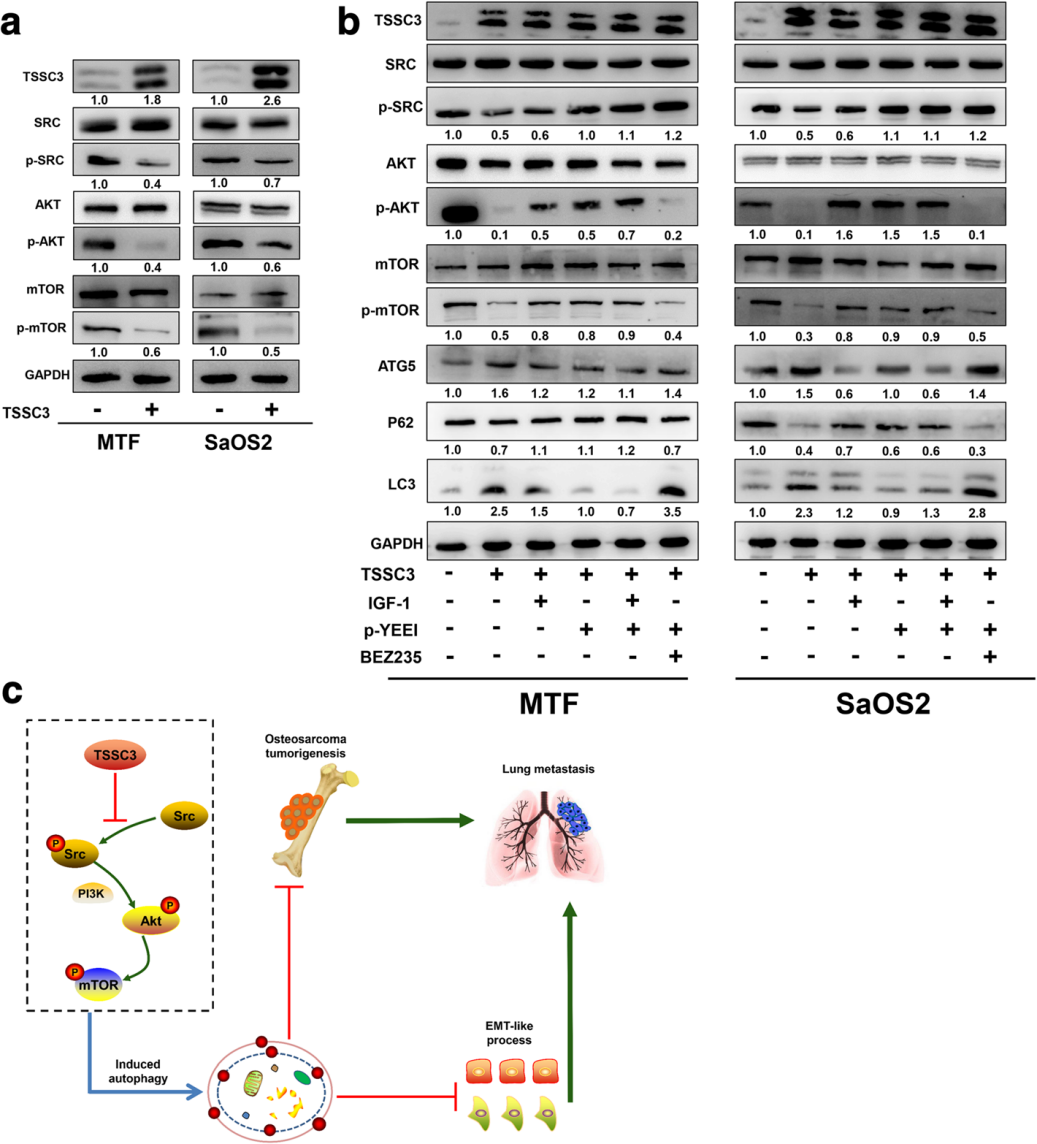
TSSC3 overexpression induces autophagy in osteosarcoma cells via inactivating the Src-mediated PI3K/Akt/mTOR pathway. **a** Representative images of the western blotting analysis of TSSC3, Src, p-Src, Akt, p-Akt, mTOR, and p-mTOR levels in MTF and SaOS2 cells. Cells were treated with or without TSSC3-overexpression. Protein levels on the western blots were quantified by densitometry of TSSC3, normalized to GAPDH and the levels of phosphorylated proteins were normalized to their total protein levels. Values are shown as fold change relative to the control (TSSC3-) groups. Quantification data was in Additional file 3: Figure S7a. **b** Representative images of the western blotting analysis of TSSC3, ATG5, P62, LC3, Src, Akt, mTOR and their phosphorylated versions in MTF and SaOS2 cells. Cells were treated with TSSC3-overexpression, IGF-1, p-YEEI, and BEZ235 separately or in combination. Protein levels on the western blots were quantified by densitometry of ATG5, LC3-II, and P62, normalized to GAPDH and the levels of phosphorylated proteins were normalized to their total protein levels. Values are shown as the fold change relative to the control groups. Quantification data was in Additional file 3: Figure S7b. **c** Schematic model illustrating how TSSC3 induces autophagy and the role of autophagy in the anti-tumor effect of TSSC3 in osteosarcoma

Our previous studies suggested TSSC3 expression might regulate the phosphorylation of Src and Akt. In view of the important role of the PI3K/Akt/mTOR pathway in autophagy regulation [27] and the role of Src in activating the PI3K/Akt pathway [11, 28], to determine how TSSC3 mediated autophagy in osteosarcoma, we first measured Src, Akt, mTOR, and their phosphorylation levels in TSSC3-overexpressing MTF and SaOS2 cells. The results showed that overexpression of TSSC3 did not affect the total Src, Akt and mTOR levels, but significantly decreased the levels of their phosphorylated forms (Fig. 7a), which suggested that the Src and PI3K/Akt/mTOR pathways might be involved in TSSC3-induced autophagy. Then, we applied IGF-1 and p-YEEI for Src and PI3K/Akt pathway activation, separately or in combination, to further identify whether the Src and PI3K/Akt/mTOR signaling pathways underlie TSSC3-mediated autophagy. TSSC3 overexpression inhibited Src and PI3K/Akt/mTOR activity, increased the levels of ATG5 and LC3-II, and decreased P62 expression. Following p-YEEI or IGF-1 treatment, the Src or PI3K/Akt/mTOR pathway was significantly activated, remarkably reversing the effect of TSSC3 overexpression on autophagy related proteins in MTF and SaOS2 cells. This implied that PI3K/Akt/mTOR or Src inhibition triggered autophagy in TSSC3-overexpressing cells (Fig. 7b). We also found that the PI3K/Akt/mTOR pathway was activated when the cells were treated with p-YEEI, but no obvious increase of Src phosphorylation was found after applying IGF-1. This result suggested that TSSC3-induced PI3K/Akt/mTOR pathway inhibition is Src-dependent. Furthermore, we applied p-YEEI and BEZ235 together to activate Src signaling and inhibit PI3K/Akt/mTOR pathway. The result indicated that inhibition of Src phosphorylation without PI3K/Akt/mTOR inactivation could not decrease the ATG5 and LC3-II levels or increase the P62 levels in TSSC3-overexpressing MTF and SaOS2 cells (Fig. 7b). Taken together, these results allowed us to conclude that TSSC3 overexpression induces autophagy in osteosarcoma cells by inactivating the Src-mediated PI3K/Akt/mTOR pathway.

## Discussion

TSSC3, a maternally expressed imprinted gene that has a potential growth inhibitory effect, was reported to loss its expression in several cancers, including osteosarcoma [8]. In our previous work, we demonstrated that *TSSC3* acts as a tumor suppressor gene in osteosarcoma [4, 8–11]. Moreover, Enhancer of zeste homolog 2 (EZH2) was found to be involved in loss of TSSC3 expression [32] and the expression of TSSC3 could be altered by 5-Aza-CdR (a DNA methyltransferase inhibitor) treatment in osteosarcoma cells [39]. Although, many anti-tumor effects were found to be associated with TSSC3 in osteosarcoma, the mechanism underlying these effects remain poorly understood. In the present study, we demonstrated that TSSC3 expression associated with ATG5 expression might be a favorable prognostic marker of osteosarcoma. We provided further evidence that TSSC3 overexpression induces autophagy, likely via the Src-mediated PI3K/Akt/mTOR pathway, and autophagy, at least partially, contributes to TSSC3-induced tumorigenesis and metastasis suppression in osteosarcoma, both in vitro and in vivo.

Accumulating evidence suggests that autophagy is correlated with progression and patient outcome after treatment in diverse types of cancer [12, 29, 36]. ATG5 and P62, as essential autophagy-related regulatory proteins, have recently been identified as novel potential prognostic biomarkers for colorectal, breast, cutaneous, and other types of cancer [38, 40–46]. However, with regard to the prognostic impact of ATG5 and P62 immunohistochemistry in various types of cancers, there are considerable discrepancies in the previous reports. For example, high expression of ATG5 suggested a superior prognosis in breast cancer, colorectal cancer, and early-stage melanoma [40–42], but indicated a poor outcome in oral squamous cell carcinoma [43]. High expression of P62 is always related to an inferior prognosis in breast cancer [44], but might lead to a longer disease-free survival in stage II melanomas [45]. Besides, these proteins may not have a prognostic value in some situations [46]. Thus, many discrepancies remain, and there are few studies on the expression dynamics of ATG5 and P62 and their prognostic roles in osteosarcoma. In the present study, we found that ATG5 was significantly downregulated, together with an accumulation of P62 in osteosarcoma tissues. This supports the notion that autophagy is a protective player in early tumorigenesis and P62 is involved in Ras-induced tumorigenesis [29, 34]. To the best of our knowledge, this is the first study to investigate the expression dynamics of ATG5 and P62 in human osteosarcoma tissues. Unfortunately, despite their notable change in expression in osteosarcoma, we failed to demonstrate an association between ATG5 or P62 with the characteristics of the patients with osteosarcoma, and neither ATG5 nor P62 seemed to be reliable prognostic biomarkers for osteosarcoma; however, this requires further validation because of our limited sample sample size. Moreover, TSSC3 was validated to be negatively associated with local recurrence and might be an independent prognostic marker for OS in osteosarcoma patients, which was similar to the observations in previous studies [11, 47]. Importantly, we demonstrated that ATG5 expression was closely and positively correlated with TSSC3 expression in osteosarcoma for the first time, indicating there was a potential correlation between TSSC3 and autophagy. More importantly, we observed that among the patients with positive TSSC3 expression, the subset with positive ATG5 expression correlated with an earlier Enneking stage and displayed a significantly better OS compared to those with negative expression of ATG5. Thus, positive ATG5 expression could be a potential predictor of favorable prognosis in patients with TSSC3(+) osteosarcoma.

In the current study, we demonstrated that TSSC3 overexpression enhanced autophagy flux in osteosarcoma cells, which was consistent with the results that ATG5 (widely known as a key protein for the formation of autophagosomes) expression was positively correlated with TSSC3 expression in human osteosarcoma tissues. *PHLDA1*, a homologous gene of *TSSC3 (PHLDA2)* has been reported to regulate autophagy in breast cancer and neuroblastoma cells [25, 48], which also suggested that TSSC3 might have the ability to regulate autophagy. As mentioned in the introduction, autophagy displays a dynamic and complicated function in cancer [21, 29, 35, 36] and can suppress tumorigenesis and metastasis in response to some stressors [2, 20, 22, 31, 37, 44]. Our findings suggested that autophagy contributes to TSSC3-mediated inhibition of tumorigenesis and metastasis in in vitro and in vivo models of osteosarcoma. EMT is widely known as an essential process during cancer progression and promotes the metastatic potential of cancer cells [38]. There is evidence that autophagy impairs the EMT by promoting Snail protein degradation [49] and weakening the stabilization of the Twist 1 protein by attenuating P62 expression [50]. Although osteosarcoma is a mesenchymal type sarcoma, we found previously that an EMT-like process exists in osteosarcoma cells that facilitates the metastatic phenotype and could be regulated by TSSC3 [4]. In the current study, our observations indicated that autophagy contributes to TSSC3-induced impairment of the EMT-like process in osteosarcoma cells. However, the mechanism by which TSSC3-induced autophagy impairs this EMT-like process in osteosarcoma cells remains unclear and further studies are need to investigate it. Autophagy can induce and contribute to apoptosis [37]. Interestingly, in our study, inhibition of autophagy inhibition displayed an opposite effect of TSSC3-induced apoptosis between MTF and SaOS2 cell lines. Similarly, autophagy inhibition was reported to have an opposing impact on the response of two osteosarcoma cell lines following camptothecin treatment [51]. Thus, we speculated that the partial autophagic effect not only varies for different cancers, but also in different cell lines.

As a member of phosphatidylinositol 3-kinase-related kinases (PIKKs), mTOR is one of the key regulators of mammalian cell metabolism and is the main downstream target of the PI3K/Akt signaling pathway [52]. Numerous reports have highlighted the importance of mTOR and its downstream target p70S6K in regulating autophagy [26, 27]. Previously, we reported that TSSC3 interacts with RanBP9 bound to Src to prevent its phosphorylation, which further abrogated Src-dependent Akt pathway activation [10]. It is reported that the homologous genes of TSSC3, for example *PHLDA1* and *PHLDA3*, are involved in the regulation of mTOR and p70S6K phosphorylation [53]. Here, we demonstrated that TSSC3 overexpression also prevented mTOR phosphorylation, and that suppression of Akt/mTOR phosphorylation is a critical factor regulating in TSSC3-induced autophagy is Src-dependent. Collectively, these findings provide a more detailed understanding of the mechanism of TSSC3’s antitumor effects (Fig. 7c).

## Conclusions

Taken together, we demonstrated, for the first time, that TSSC3 induces autophagy via inhibiting the Src-dependent PI3K/Akt/mTOR pathway in osteosarcoma. Furthermore, we found that TSSC3-induced autophagy contributes to suppressing tumorigenesis and metastasis in osteosarcoma, both in vitro and in vivo. In addition, TSSC3 is an independent prognostic marker for OS in patients with osteosarcoma, and TSSC3-associated positive ATG5 expression might be a potential applicable predictor of favorable prognosis. Therefore, our results highlight the importance of autophagy in TSSC3-induced antitumor effects and provide important insights into the underlying mechanism of TSSC3-induced antitumor effects.

## Additional files

Additional file 1: Supplementary Materials and Methods. (RTF 91 kb)

Additional file 2: Supplementary Tables. **Table S1.** shRNA target sequence used in the experiments. **Table S2.** Primers used for qRT-PCR in this study. **Table S3.** Correlation between TSSC3 expression and ATG5 or P62 expression in patients with osteosarcoma. **Table S4.** Univariate Cox proportional hazard regression analysis for overall survival in patients with osteosarcoma. **Table S5.** Correlations between ATG5 expression and the metastasis or recurrence of positive-TSSC3 expression osteosarcoma. (RTF 137 kb)

Additional file 3: Supplementary Figures. **Figure S1.** Immunohistochemistry staining of TSSC3, ATG5 and P62 in human benign bone and soft tissue tumors and osteosarcoma tissues. **Figure S2.** TSSC3 overexpression enhances ATG5 and BECN1 expression and the block autophagic flux function of chloroquine. **Figure S3.** TSSC3 overexpression inhibited cells growth *in vitro* and autophagy suppression attenuates OverTSSC3-induced apoptosis in SaOS2 cells but not in MTF cells. **Figure S4.** Downregulation of ATG5 restrained the autophagy promotion and reversed the inhibition of osteosarcoma tumorigenicity caused by TSSC3 overexpression *in vivo*. **Figure S5.** The quantification of western blot results in Figure 5e and f. **Figure S6.** Immunocytochemical analysis of metastatic lung nodules and Kaplan–Meier overall survival curves from the *in vivo* metastasis model. **Figure S7.** The quantification of western blot results in Figure 7. (DOCX 7590 kb)

## Abbreviations

*ATG5*: Autophagy-related gene 5 (*ATG5*)
AVs: **A**utophagic vesicles
*BECN1*: Beclin1, autophagy-related gene 6
**CQ**: **C**hloroquine
EMT: Epithelial-mesenchymal transition
IHC: Immunohistochemistry
mTOR: Mammalian target of rapamycin
OS: Overall survival
PHLDA2: Pleckstrin homology-like domain, family A, member 2
*TSSC3*: Tumor-suppressing STF cDNA 3
