# Supplementary material for: TSSC3 promotes autophagy via inactivating the Src-mediated PI3K/Akt/mTOR pathway to suppress tumorigenesis and metastasis in osteosarcoma, and predicts a favorable prognosis
Source: J Exp Clin Cancer Res. 2018 Aug 9;37:188. doi: 10.1186/s13046-018-0856-6 (PMC6085607; doi:10.1186/s13046-018-0856-6)
Supplement: Supplementary file 1 — Supplementary Materials and Methods. (RTF 91 kb) [file 13046_2018_856_MOESM1_ESM.rtf]

Additional file 1

SUPPLEMENTARY MATERIALS AND METHODS
Human specimens
Human benign bone and soft tissue tumor specimens were obtained from 21 patients with histopathologically-confirmed fibrous dysplasia and 12 patients with histopathologically-confirmed osteoblastoma from Xinqiao Hospital, Third Military Medical University (TMMU), Chongqing, China from 2015 to 2017. Written informed consent for the experimental studies was obtained from the patients or their guardians. All experiments were approved by the Institutional Ethics Committee of TMMU.
Western blotting analysis
Proteins were separated using 8–12% SDS Tris-glycine gels and transferred onto PVDF membranes (3010040001, Roche, Shanghai, China). Membranes were blocked with 5% fat-free milk and incubated with the appropriate primary antibodies overnight at 4 °C. Antibodies against GAPDH (1:1000, AB-P-R001) were purchased from Hangzhou Goodhere Biotechnology Co., Ltd (Hangzhou China). ATG5 (1:1000, 12994T), E-cadherin (1:300, 3195T), N-cadherin (1:1000, 13116T), Vimentin (1:1000, 5741S), LC3B (1:1500, 3868T), MMP2 (1:500, 40994S), p-mTOR (1:1000, 5536T), SRC (1:1000, 2019S), p-SRC (1:1000, 6943S), AKT (1:1000, 4691T), p-AKT (1:1000, 4060S), cleaved Caspase3 (1:1000, 9664T), BCL2 (1:1000, 2872T), and Bax (1:1000, 5023T) antibodies were purchased from Cell Signaling Technology (CST, Danvers, MA, USA); BECN1 (1:2000, ab207612), m-TOR (1:1000, ab) antibodies were purchased from Abcam (Cambridge, MA, USA). TSSC3 (1:1000, 14661-1-AP) antibodies were purchased from Proteintech Group, Inc (Proteintech, Wuhan, China) and P62 (1:500, GTX100685) antibodies were purchased from GeneTex (Alton Pkwy Irvine, CA, USA). Then, the secondary antibody (goat anti-rabbit IgG, Bioss, Beijing, China) was applied. Immunoreactivity was detected using an ECL Kit (BeyoECL Moon, Beyotime, Shanghai, China). 
Immunofluorescence
The cells infected with overCtrl or OverTSSC3 Lentivirus were plated onto 35 mm glass bottom culture dishes (NEST Biotechnology, Wuxi, China) in Dulbecco's modified Eagle's medium (DMEM) with 10% fetal bovine serum (FBS) for 24 h. After that, the cells were incubated in 4% paraformaldehyde in phosphate-buffered saline (PBS) for 20 min at room temperature, permeabilized with 0.1% Triton X-100 (Solarbio, Beijing, China) in PBS for 15 min, and then blocked with 5% bovine serum albumin and incubated with primary antibody against TSSC3 (1:100, Abcam), ATG5 (1:100, Abgent), LC3B (1:200, CST) or BECN1 (1:100) overnight at 4 °C and then with fluorescent secondary antibodies (Goat Anti-rabbit IgG/Alexa Fluor 555, Bioss, Beijing, China or Goat Anti-rabbit IgG/Alexa Fluor 647, Beyotime, Shanghai, China) for 2 h at room temperature. Hoechst 33258 was used to counterstain nuclei for 6 min at room temperature. The images were obtained using a confocal microscope (Carl Zeiss LSM510, Germany) and examined using Leica LAS-AF Lite software.
Apoptosis analysis
Cell apoptosis was analyzed by Hoechst staining and Annexin V-PE/7-ADD analysis. After the cells were infected with TSSC3 overexpression, or treated with chloroquine (CQ) (8 µM for 12 h), or a combination of both, the cells seeded in 24-well plates, fixed in 4% paraformaldehyde for 20 min, and then stained with Hoechst 33258 for 15 min. Nuclear morphology was viewed using an inverted phase contrast fluorescence microscope (Olympus, Tokyo, Japan). The number of cells with apoptotic morphology (appearing condensed or with fragmented nuclei) was counted in four random fields, and included over 50 cells per experimental group. Annexin V-PE/7-ADD analysis was performed by using an Annexin V-PE/7-ADD kit (Becton Dickinson, BD, Franklin Lakes, NJ, USA) according to the manufacturer's protocols. Briefly, harvested cells were washed with PBS and then resuspended in 200 µl binding buffer. Subsequently, a total of 5 µl of Annexin V-PE and 5 µl 7-ADD were added and incubated for 15 min at room temperature in the dark. Flow cytometric analysis was performed using a FACSCalibur software (BD, NJ, USA). Annexin V-PE positive, 7-ADD negative cells were identified as early apoptotic cells. Annexin V-PE and 7-ADD positive cells were identified as late apoptotic cells.
Histology and immunohistochemistry
Immunohistochemistry (IHC) was performed using an IHC kit (Zsbio, Beijing, China). Sample sections were deparafinized through a series of xylene baths, antigens were retrieved by steam treatment in 10 mM citrate buffer, blocked with 3% hydrogen peroxide for 15 min at 37 °C, pre-incubated with blocking serum solution for 30 min at 37 °C, and then incubated at 4 °C with the primary antibodies overnight. Subsequently, the secondary antibodies were applied and the nuclei were counterstained with hematoxylin. The slides were then examined from non-overlapping cells using a light microscope (Olympus, Tokyo, Japan). Primary rabbit anti-human TSSC3 (1:30, Proteintech, Wuhan, China), rabbit anti-human ATG5 (1:150, Abgent), rabbit anti-human P62 (1:200, GeneTex), rabbit anti-human LC3B (1:150, CST) and ready-to-use mouse anti-human Ki67, E-cadherin, CK18, and Vimentin (MXB Biotechnologies, Fuzhou, China) were used. Specimens were independently scored by two pathologists who were blinded to the clinical and pathological data. For semi-quantitative assessment of protein expression, the percentage of positive cells was calculated in more than five randomly selected fields of view using higher-magnification objectives (× 400), and included over 50 cells. The final IHC score was a product of the positive cell ratio score (0 = no immunoreactivity; 1 ≤ 25% cells stained; 2 = 26–50% cells stained; 3 = 51–75% cells stained; and 4 = ≥76% cells stained) and relative expression score (0, negative; 1, yellow staining; 2, brown staining; 3, dark brown staining). Final scores ≥ 2 were considered positive expression for TSSC3 (average score was 1.38) while for ATG5 (average score was 3.67) and P62 (average score was 4.21) scores ≥ 4 were considered positive expression.
