# Supplementary material for: TSSC3 promotes autophagy via inactivating the Src-mediated PI3K/Akt/mTOR pathway to suppress tumorigenesis and metastasis in osteosarcoma, and predicts a favorable prognosis
Source: J Exp Clin Cancer Res. 2018 Aug 9;37:188. doi: 10.1186/s13046-018-0856-6 (PMC6085607; doi:10.1186/s13046-018-0856-6)
Supplement: Supplementary file 2 — Supplementary Tables. Table S1. shRNA target sequence used in the experiments. Table S2. Primers used for qRT-PCR in this study. Table S3. Correlation between TSSC3 expression and ATG5 or P62 expression in patients with osteosarcoma. Table S4. Univariate Cox proportional hazard regression analysis for overall survival in patients with osteosarcoma. Table S5. Correlations between ATG5 expression and the metastasis or recurrence of positive-TSSC3 expression osteosarcoma. (RTF 137 kb) [file 13046_2018_856_MOESM2_ESM.rtf]

Additional file 2
SUPPLEMENTARY TABLES
Table S1. shRNA target sequence used in the experiments.
ShRNA 	target sequence (5′-3′)	
Scrambled	TTCTCCGAACGTGTCACGTAA	
ShATG5-1	CCTATGAATTCAAGAGTACTGTGGA	
ShATG5-2	GATTCATGGAATTGAGCCAAT	


Table S2. Primers used for qRT-PCR in this study.
Target gene	Forward primer sequence (5′-3′)	Reverse primer sequence (5′-3′)	
GAPDH	GGAGCGAGATCCCTCCAAAAT	GGCTGTTGTCATACTTCTCATGG	
TSSC3	TCCAGCTATGGAAGAAGAAGC	GTGGTGACGATGGTGAAGTACA	
ATG5	TCAGCCACTGCAGAGGTGTTT	GGCTGCAGATGGACAGTTGCA
	
BECN1	CTCTCGCAGATTCATCCCCC
	GACGTTGAGCTGAGTGTCCA
	
The primers were all obtained from Sangon Biotech (Sangon, Shanghai, China).


Table S3. Correlation between TSSC3 expression and ATG5 or P62 expression in patients with osteosarcoma.
TSSC3	n	ATG5	P62 	
		Positive	Negative	Positive	Negative	
Positive	17	14	3	9	8	
Negative	41	16	25	32	9	
r		0.395	-0.251	
P		0.003*	0.067	

r Spearman's correlation analysis.
* p < 0.05


Table S4. Univariate Cox proportional hazard regression analysis for overall survival in patients with osteosarcoma.
Variable
No.
Hazard ratio
(95% Confidence interval)
p

Age ≥ 21 y (vs. < 21 y)


25/58
1.167
(0.545 to 2.498)

0.690

Gender Male (vs. Female)

34/58
1.579
(0.742 to 3.362)

0.236

Enneking stage III (vs. II)

12/58
4.349
(1.993 to 9.492)

<0.001*

Tumor size, cm, ≥ 8 cm
(vs. < 8 cm)

17/58
2.996
(1.373 to 6.539)

0.006*

Local recurrence (yes vs. no)

22/58
2.631
(1.219 to 5.677)

0.014*

Lung metastasis (yes vs. no)

20/58
7.080
(3.116 to 16.086)

<0.001*

TSSC3
negative expression 
(vs. positive expression)

41/58
4.776
(1.434 to 15.906)

0.011*

ATG5 
negative expression 
(vs. positive expression)

28/58
1.795
(0.835 to 3.861)

0.134

P62
positive expression 
(vs. negative expression)

41/58
1.431
(0.605 to 3.387)

0.415


*with significant difference (P < 0.05)


Table S5. Correlations between ATG5 expression and the metastasis or 
recurrence of positive-TSSC3 expression osteosarcoma.
Clinical 
characteristics
Group
n
ATG5
Positive  

negative
P-value#

Lung metastasis
Yes
3
1
2
0.063


No
14
13
1


Local recurrence
Yes
3
2
1
0.465


No
14
12
2


Stage
IIA+IIB
15
14
1
0.022*


III
2
0
2


££Fisher's exact test, *with significant difference (P < 0.05)


	
