# Supplementary material for: TSSC3 promotes autophagy via inactivating the Src-mediated PI3K/Akt/mTOR pathway to suppress tumorigenesis and metastasis in osteosarcoma, and predicts a favorable prognosis
Source: J Exp Clin Cancer Res. 2018 Aug 9;37:188. doi: 10.1186/s13046-018-0856-6 (PMC6085607; doi:10.1186/s13046-018-0856-6)
Supplement: Supplementary file 3 — Supplementary Figures. Figure S1. Immunohistochemistry staining of TSSC3, ATG5 and P62 in human benign bone and soft tissue tumors and osteosarcoma tissues. Figure S2. TSSC3 overexpression enhances ATG5 and BECN1 expression and the block autophagic flux function of chloroquine. Figure S3. TSSC3 overexpression inhibited cells growth in vitro and autophagy suppression attenuates OverTSSC3-induced apoptosis in SaOS2 cells but not in MTF cells. Figure S4. Downregulation of ATG5 restrained the autophagy promotion and reversed the inhibition of osteosarcoma tumorigenicity caused by TSSC3 overexpression in vivo. Figure S5. The quantification of western blot results in Figure 5e and f. Figure S6. Immunocytochemical analysis of metastatic lung nodules and Kaplan–Meier overall survival curves from the in vivo metastasis model. Figure S7. The quantification of western blot results in Figure 7. (DOCX 7590 kb) [file 13046_2018_856_MOESM3_ESM.docx]

**Additional file 3**

**SUPPLEMENTARY FIGURES**


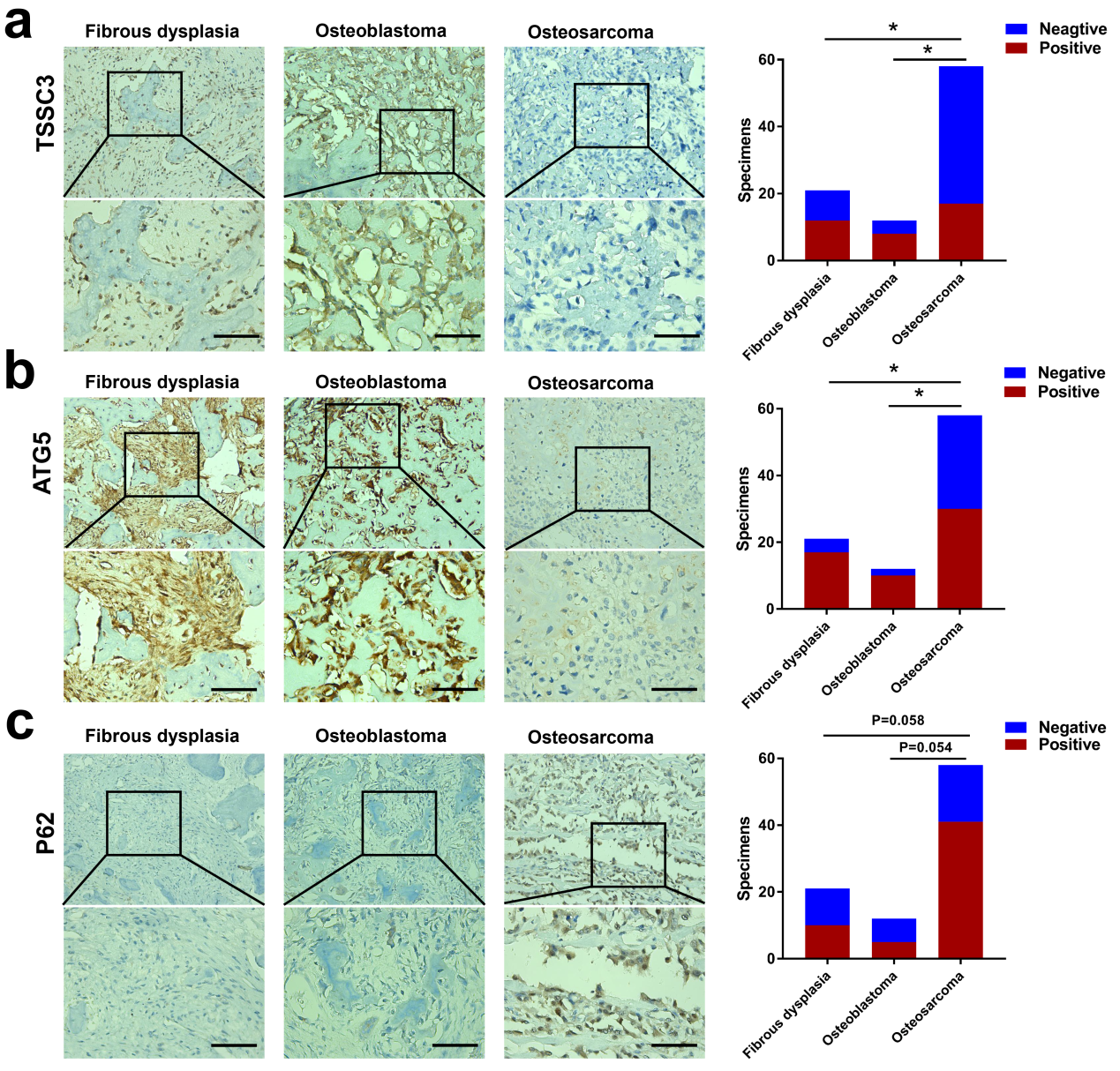


**Figure S1. Immunohistochemistry staining of TSSC3, ATG5 and P62 in human benign bone and soft tissue tumors and osteosarcoma tissues.**

**a-c** Representative images of TSSC3, ATG5 and P62 expression in osteoblastoma, fibrous dysplasia and osteosarcoma. TSSC3 and ATG5 expression are notably higher in fibrous dysplasia and osteoblastoma than osteosarcoma, while P62 expression is no statistical significance lower in fibrous dysplasia and osteoblastoma than osteosarcoma. Quantitative comparison of TSSC3, ATG5 and P62 positive expression rate between osteosarcoma and benign bone and soft tissue tumor are also given.

Significant differences were determined using Chi-squared test. *: *P*<0.05. Scale bars: 50um.

**
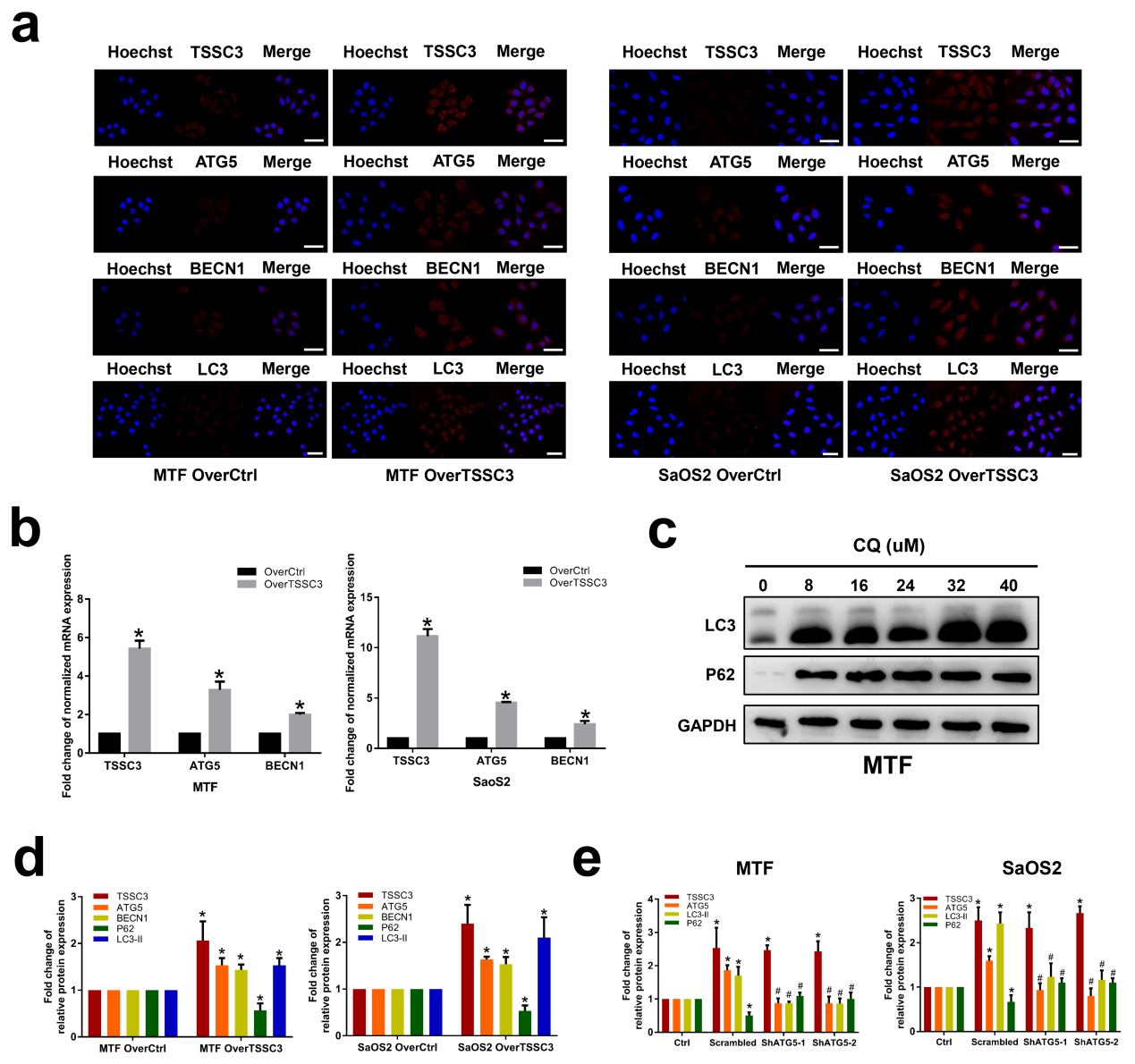
**

**Figure S2. TSSC3 overexpression enhances ATG5 and BECN1 expression and the block autophagic flux function of chloroquine.**

**a** TSSC3 over expression enhances ATG5 and BECN1 expression in MTF and SaOS2 cells detected by Immunofluorescence analysis.

**b** TSSC3 over expression enhances ATG5 and BECN1 expression detected by RT-qPCR. Expression of mRNA were normalized to GAPDH and showed as a fold change to the OverCtrl groups. Significant differences were determined using unpaired Student’s t test. *: P<0.05 compared to OverCtrl groups. Scale bars: 50um.

**c** Western blotting of LC3 and p62 in MTF cells treated with chloroquine (CQ) as the indicated concentration for 12 h. LC3-II and p62 protein expression showed an notably increase with the presence of autophagy flux inhibitors chloroquine (8 uM) and no visible difference with a higher concentration.

**d** The quantification of western blot results in Figure 2 b. Significant differences were determined using one-way ANOVA with Bonferroni’s multiple comparisons. *: *P*<0.05, compared to Ctrl group.

**e** The quantification of western blot results in Figure 3 d. Significant differences were determined using one-way ANOVA with Bonferroni’s multiple comparisons. *: *P*<0.05, compared to Ctrl group; #: P<0.05, compared to Scrambled group.

**
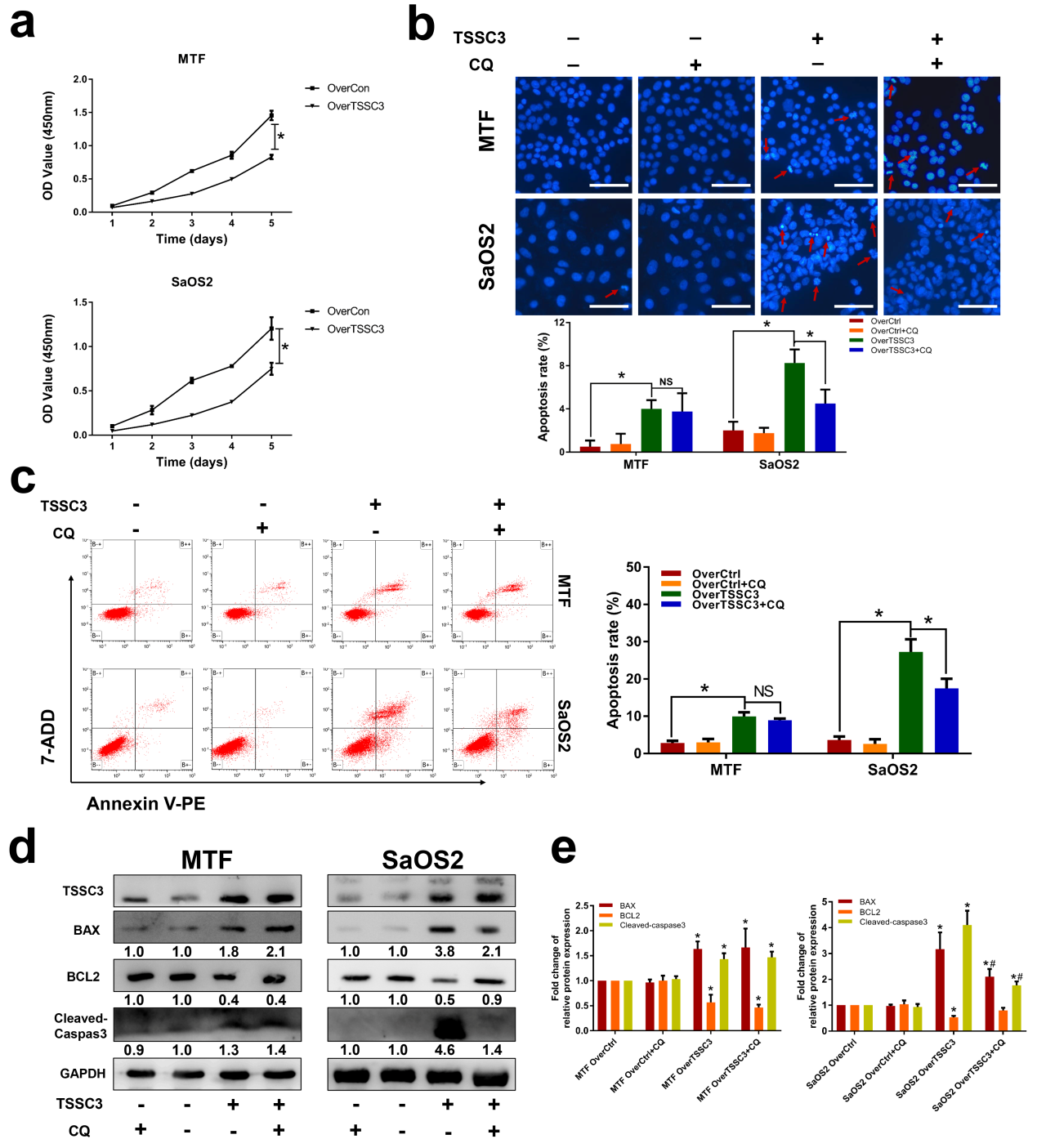
**

**Figure S3. TSSC3 overexpression inhibited cells growth *in vitro* and autophagy suppression attenuates OverTSSC3-induced apoptosis in SaOS2 cells but not in MTF cells.**

**a** The cell viability was determined by CCK-8 treated with or without TSSC3 overexpression for 5 days. Cells were plated in 96-well plates and 10^3^ cells for each well. Significant differences were determined using Student’s t test.

**b**  Hoechst 33258 stain was used to identify apoptosis in cells infected with TSSC3 overexpression, or CQ (8 µM for 12 h), or a combination of both. Red arrows indicated representative apoptosis cells. Significant differences were determined using one-way ANOVA with Bonferroni’s multiple comparisons.

**c**  MTF and SaOS2 cells were treated as in **b**, and cell apoptosis was analysed by flow cytometry with Annexin V-PE/7-AAD. The apoptotic rate is shown from triplicate experiments. Bar graph of apoptosis (right) rate is shown. Significant differences were determined using one-way ANOVA with Bonferroni’s multiple comparisons.

**d** TSSC3, BAX, BCL2, and cleaved-Caspase 3 protein levels were detected by Western blot in cells treated with TSSC3 overexpression, or CQ (8 µM for 12 h), or a combination of both. GAPDH: internal control. Western blot values were normalized to GAPDH and presented as a fold change of the control group.

**e** The quantification of western blot results in Figure S3 d.

*: P<0.05, NS: No Significance. Scale bars: 50um.


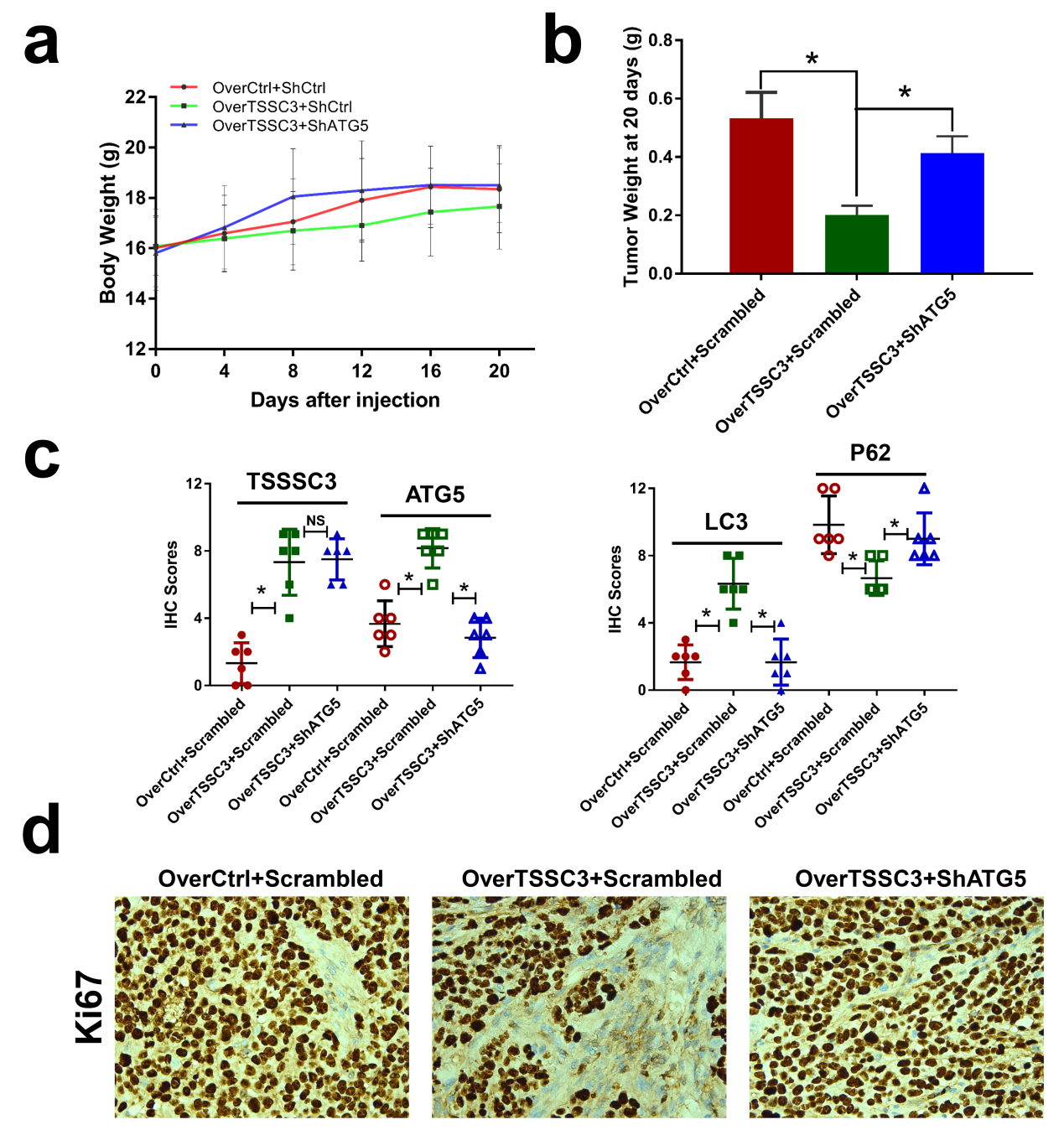


**Figure S4. Downregulation of ATG5 restrained the autophagy promotion and reversed the inhibition of osteosarcoma tumorigenicity caused by TSSC3 overexpression *in vivo*.**

**a-b** Body weights and tumor weights were measured.

**c** The IHC scores quantification of TSSC3, ATG5, LC3 and P62 expression levels in osteosarcoma *in vivo* after infected with OverTSSC3 or OverTSSC3 and ShATG5 as indicated (n=6).

**d** Representative images of Ki67 after treatment as indicated *in vivo*.

Significant differences were determined using one-way ANOVA with Bonferroni’s multiple comparisons. *: *P*<0.05, NS: No Significance.


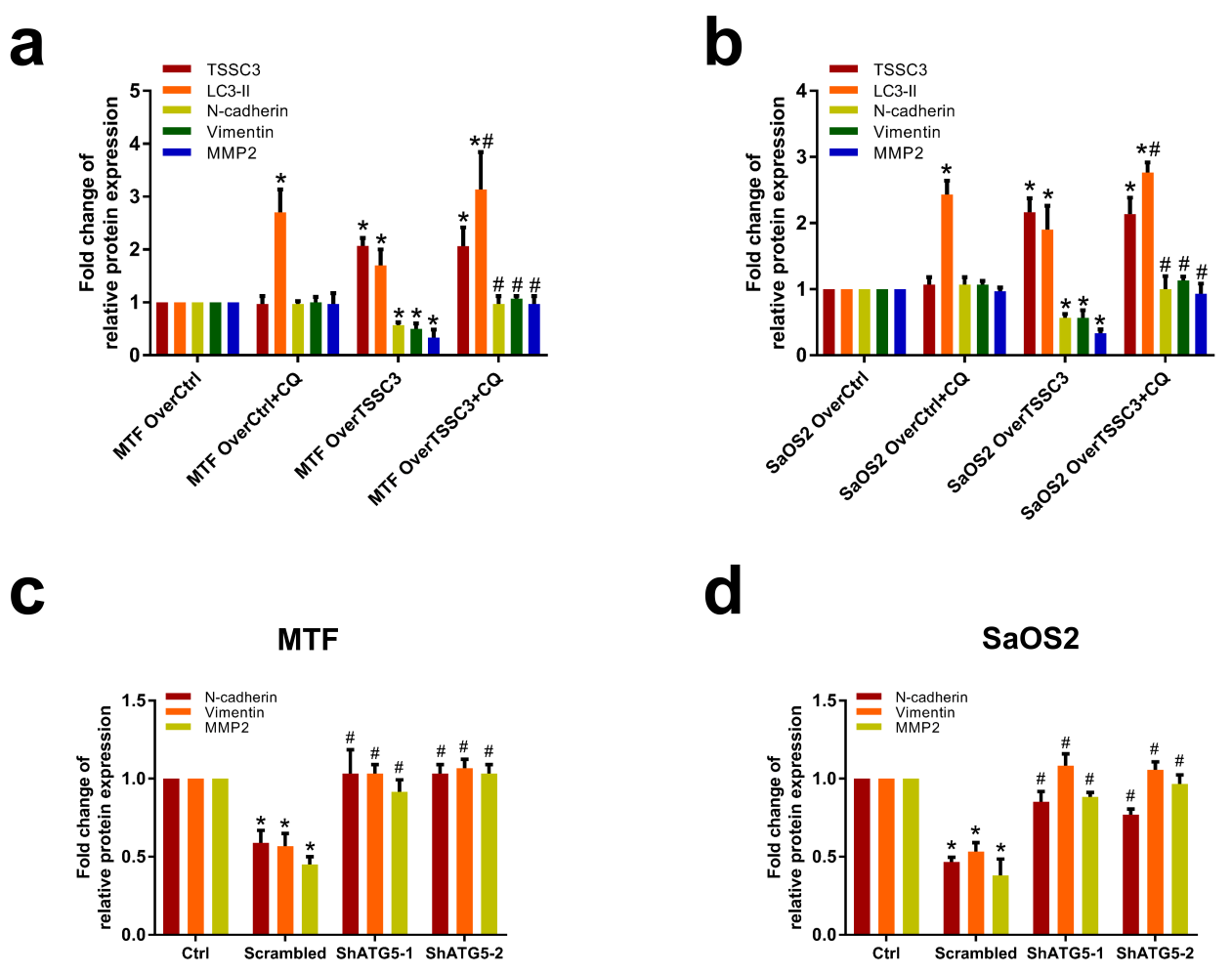


**Figure S5. The quantification of western blot results in Figure 5 e and Figure 5 f.**

**a-b** The quantification of western blot results in Figure 5 e. Significant differences were determined using one-way ANOVA with Bonferroni’s multiple comparisons. *: *P*<0.05, compared to Ctrl group; #: P<0.05, compared to OverTSSC3 group.

**c-d** The quantification of western blot results in Figure 5 f. Significant differences were determined using one-way ANOVA with Bonferroni’s multiple comparisons. *: *P*<0.05, compared to Ctrl group; #: P<0.05, compared to Scrambled group.


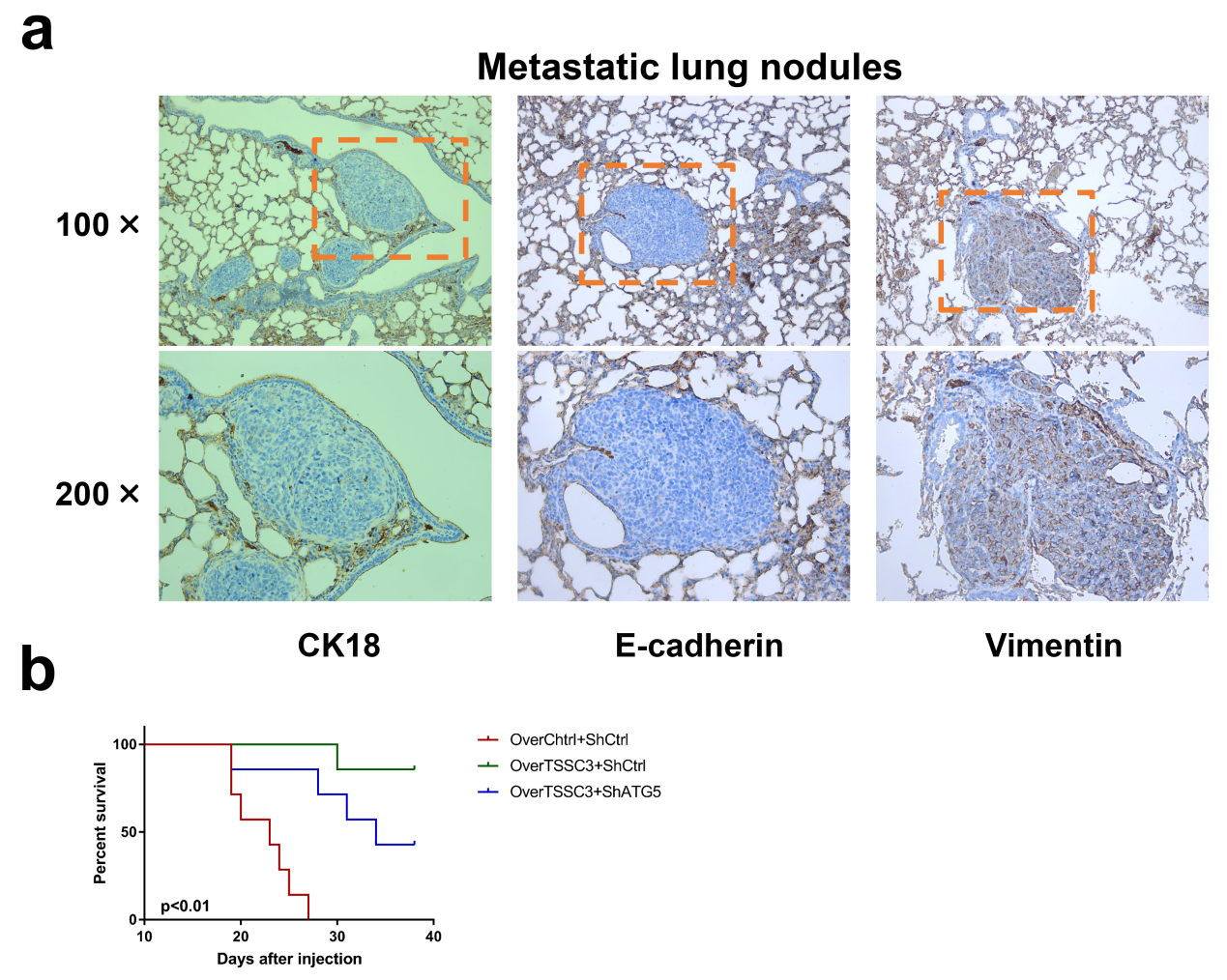


**Figure S6. Immunocytochemical analysis of metastatic lung nodules and Kaplan–Meier overall survival curves from the *in vivo* metastasis model.**

**a** Immunocytochemical analysis of E-cadherin, Vimentin and CK18 in metastatic lung nodules *in vivo* metastasis model.

**b** Kaplan–Meier overall survival curves from the *in vivo* metastasis model for 38 days after injection.

Significant differences were determined using the log-rank test.


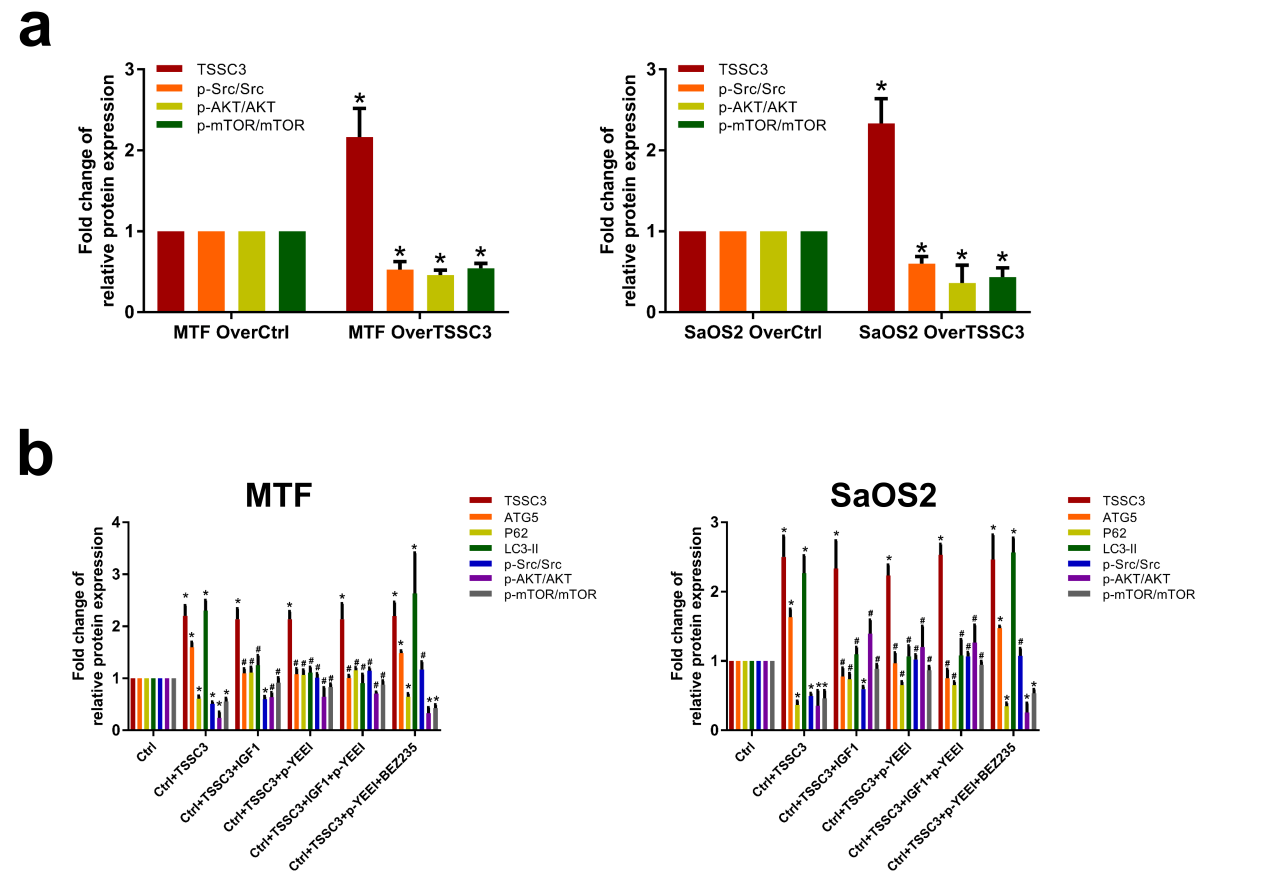


**Figure S7. The quantification of western blot results in Figure 7.**

**a** The quantification of western blot results in Figure 7 a. Significant differences were determined using one-way ANOVA with Bonferroni’s multiple comparisons. *: *P*<0.05, compared to Ctrl group.

**b** The quantification of western blot results in Figure 7 b. Significant differences were determined using one-way ANOVA with Bonferroni’s multiple comparisons. *: *P*<0.05, compared to Ctrl group; #: P<0.05, compared to OverTSSC3 group.
